# Genome wide comparison of Ethiopian *Leishmania donovani* strains reveals differences potentially related to parasite survival

**DOI:** 10.1371/journal.pgen.1007133

**Published:** 2018-01-09

**Authors:** Arie Zackay, James A. Cotton, Mandy Sanders, Asrat Hailu, Abedelmajeed Nasereddin, Alon Warburg, Charles L. Jaffe

**Affiliations:** 1 Dept Microbiology & Molecular Genetics, The Kuvin Center for the Study of Infectious & Tropical Diseases, IMRIC, Hebrew University–Hadassah Medical School, Jerusalem, Israel; 2 Wellcome Trust Sanger Institute, Wellcome Trust Genome Campus, Hinxton, United Kingdom; 3 Dept Microbiology, Immunology & Parasitology, Addis Ababa University, Addis Ababa, Ethiopia; Princeton University, UNITED STATES

## Abstract

*Leishmania donovani* is the main cause of visceral leishmaniasis (VL) in East Africa. Differences between northern Ethiopia/Sudan (NE) and southern Ethiopia (SE) in ecology, vectors, and patient sensitivity to drug treatment have been described, however the relationship between differences in parasite genotype between these two foci and phenotype is unknown. Whole genomic sequencing (WGS) was carried out for 41 *L*. *donovani* strains and clones from VL and VL/HIV co-infected patients in NE (n = 28) and SE (n = 13). Chromosome aneuploidy was observed in all parasites examined with each isolate exhibiting a unique karyotype. Differences in chromosome ploidy or karyotype were not correlated with the geographic origin of the parasites. However, correlation between single nucleotide polymorphism (SNP) and geographic origin was seen for 38/41 isolates, separating the NE and SE parasites into two large groups. SNP restricted to NE and SE groups were associated with genes involved in viability and parasite resistance to drugs. Unique copy number variation (CNV) were also associated with NE and SE parasites, respectively. One striking example is the folate transporter (FT) family genes (*LdBPK*_100390, *LdBPK*_100400 and *LdBPK*_100410) on chromosome 10 that are single copy in all 13 SE isolates, but either double copy or higher in 39/41 NE isolates (copy number 2–4). High copy number (= 4) was also found for one Sudanese strain examined. This was confirmed by quantitative polymerase chain reaction for *LdBPK_100400*, the *L*. *donovani* FT1 transporter homolog. Good correlation (p = 0.005) between FT copy number and resistance to methotrexate (0.5 mg/ml MTX) was also observed with the haploid SE strains examined showing higher viability than the NE strains at this concentration. Our results emphasize the advantages of whole genome analysis to shed light on vital parasite processes in *Leishmania*.

## Author summary

Approximately 200,000–400,000 new cases of visceral leishmaniasis (VL) occur annually resulting in an estimated 40,000 deaths. Almost 90% of the reported cases are caused by the *Leishmania donovani* occurring primarily in East Africa and the Indian subcontinent. Parasites in East Africa are more polymorphic than those isolated in other regions, and differences in vectors, biotopes and patient response to drugs are found in Ethiopia. Large-scale whole genome sequence (WGS) analysis of *L*. *donovani* strains and clones isolated from VL and HIV^+^-VL co-infected patients in Ethiopia was carried out. Single nucleotide polymorphism (SNP) and gene copy number variation (CNV) analysis shows genetic differences correlated with geographic regions in Ethiopia. These differences are associated with distinct biological processes and molecular functions, and may be associated with genes involved in drug resistance and parasite survival.

## Introduction

*Leishmania donovani*, together with *L*. *infantum*, are the main causative agents of visceral leishmaniasis (VL). The World Health Organization (WHO) estimates that this disease causes an estimated 200,000 to 400,000 new VL cases worldwide, and >40,000 deaths yearly. The majority of VL cases occur on the Indian subcontinent, Brazil, and East Africa with most cases in the latter region found in Sudan, South Sudan and Ethiopia [1]. While treatment regimens for VL, including combination therapy based on existing drugs, have improved safety and prognosis, they are still suboptimal, and new drugs are urgently needed. High parasite resistance on the Indian subcontinent to the pentavalent antimonial sodium stibogluconate (SSG) has led to its discontinuation, however SSG is still part of the primary treatment regimen for VL in Ethiopia. The situation in Ethiopia is further complicated by the presence of AIDS, a leading cause of adult illness and death in this country [2, 3]. Between 20–40% of VL patients are co-infected with HIV and relapses, up to 50% at a year post-treatment, are frequently observed [2, 4]. Treatment following relapse normally utilizes alternative drugs like liposomal amphotericin B, pentamidine and paromomycin. Paromomycin is an antibiotic recently approved to treat VL in India and is in clinical trials in Africa [5]. Differences in the dose of paromomycin required to treat VL patients from northern Ethiopia (NE) and Sudan, as compared to southern Ethiopia (SE) and Kenya have been reported [5, 6]. Interestingly, other differences in the ecology, sand fly vectors and parasites have been described between NE and SE. Endemic regions of NE are typically semi-arid, with commercial monoculture fields and scattered Acacia–Balanite forests [7–9]. *Phlebotomus orientalis* is the primary vector in this region. On the other hand, transmission in SE occurs in areas of savannah and forest where termite mounds abound; and *Phlebotomus martini* and *Phlebotomus celiae* have been implicated as vectors [7, 8, 10, 11]. Molecular characterization including multilocus enzyme electrophoresis (MLEE), multilocus microsatellite typing (MLMT), protein and DNA sequence analysis of individual genes, and k26—PCR targeting the hydrophilic acylated surface protein B (HASPB) repeat region have been used to characterize the *L*. *donovani* complex in East Africa [7, 12–15]. MLMT analysis indicated that different genetic populations and subpopulations are present in NE and SE [13].

Recent developments in whole genome sequencing (WGS) and computational analysis allow the in-depth exploration and comparison of leishmanial genomes at a high level of resolution and accuracy [16–18]. Genomes of individual *Leishmania* species [18]; of *L*. *infantum* or *L*. *donovani* strains in limited geographic regions of Turkey or India/Nepal respectively [16, 19, 20], and strains showing differences in drug resistance and tropism [16, 20–24] have been analyzed by WGS. In this report 41 patient strains and clones from NE and SE are analyzed and compared by WGS. This study provides insights into the population structure, and genetic differences of parasites circulating in the distinct ecologies of Ethiopia. The evidence of genomic variation between the two *L*. *donovani* populations (NE and SE) may provide an additional insight about parasite virulence, development of drug resistance and give new directions for new treatment strategies.

## Results

WGS of 41 *L*. *donovani* parasites, both clones and isolates, from 15 Ethiopian patients was carried out and analyzed (S1 Table). All 18 patient strains were isolated in the years 2009 and 2010 in NE (n = 9) or SE (n = 10). The parasites analyzed were isolated from spleens, bone marrows and skin lesions. Five of the patients had AIDS at the time the parasites were isolated; and in three cases, two strains obtained from different organs (either spleen and skin, or spleen and bone marrow), were analyzed. None of the patients received treatment for VL prior to parasite isolation. A Sudanese strain, isolated in 1998, was also included in some analysis (S1 Table).

### Aneuploidy

**Fig 1:**
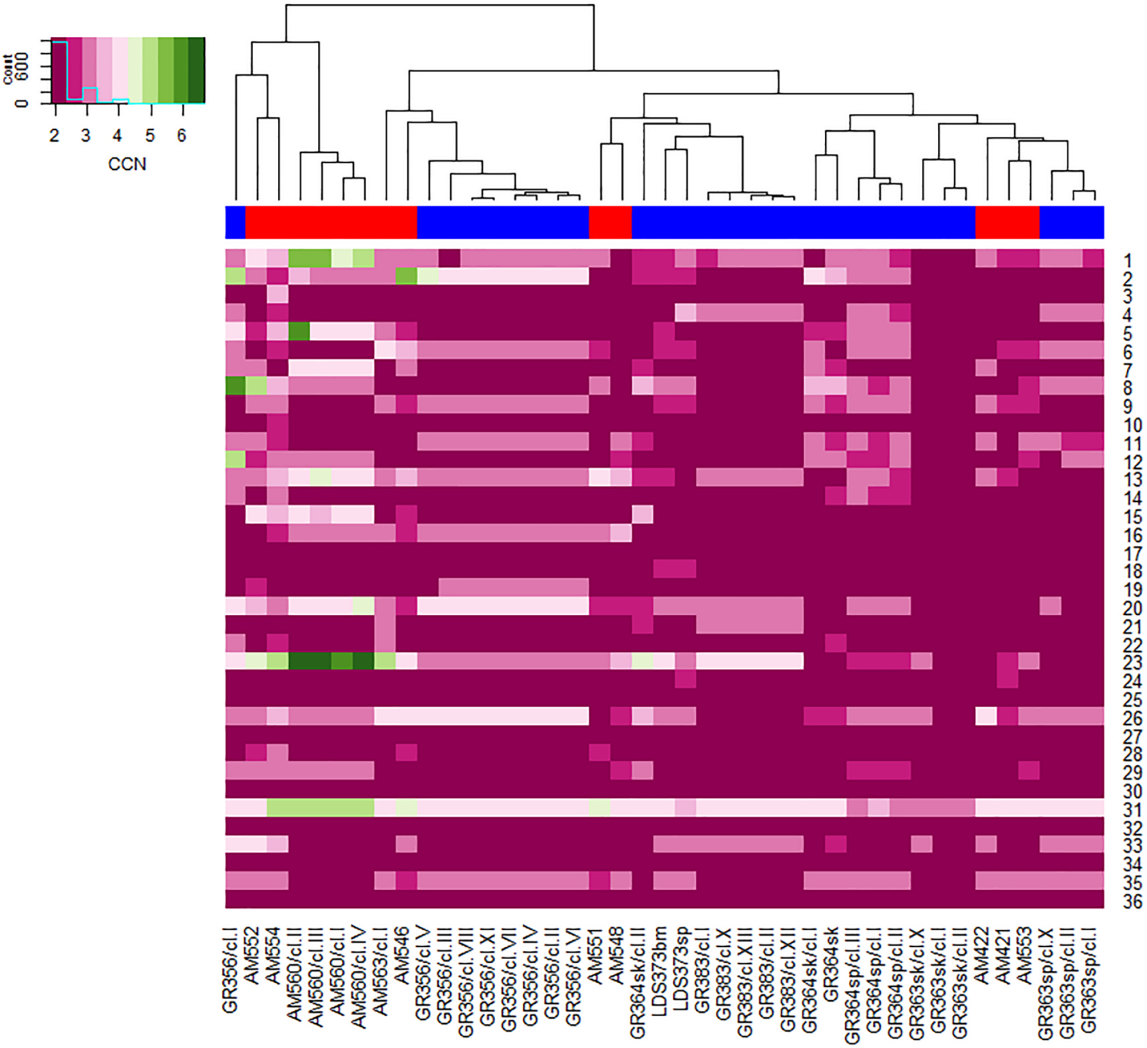
Comparison of aneuploidy profiles of *Leishmania donovani* from northern and southern Ethiopia. Chromosome numbers are listed down the right side of the heat map. Across the bottom the *L*. *donovani* isolates, clones and parental patient strains, analyzed are indicated. The dendrogram on the top shows clusters of the strains and clones based on similarity of aneuploidy profiles and was generated by comparison of the Euclidean distances between aneuploidy profiles among strains (R packages; *stats*, *gplots*). Bar under dendrogram indicates the parasite source: Red–southern Ethiopia and Blue–northern Ethiopia. Insert: color key indicating ploidy of chromosomes in heat map. The minimum number of chromosomes, 2, is diploid. Green indicates chromosomal copy number > 4 and pink ≤ 4.

Chromosome copy number was predicted based on whole chromosome median read coverage as described in Material and Methods. The predicted values for each chromosome in the patient isolates and their clones are given in Fig 1 and S2 Table. Normalized read depth, showed that 68.5% of the chromosomes had a predicted chromosome (PCHR) copy number of 2 ± 0.5, i.e. disomic, with smaller percentages showing higher copy numbers; i.e., trisomic (3 ± 0.5) in 23.4%, tetrasomic (4 ± 0.5) in 6.7%, pentasomic (5 ± 0.5) in 1% and hexasomic (6 ± 0.5) in 0.4%, similar to what was previously reported for Nepalese *L*. *donovani* strains [16]. Several strains, mostly patient isolates and clones belonging to the SE population; show intermediate ploidy (mixoploidy) for specific chromosomes, e.g. chromosomes 1, 6 and 23 (S2 Table), suggestive of parasite populations with mixed polysomic diversity, perhaps due to variation in chromosomal amplification between individual cells in culture. Differences in chromosome aneuploidy between SE patient strain and its clones was not significant (unpaired two samples student's t-test, p = 0.2), suggesting that aneuploidy in the clonal population is not due to selection of clones exhibiting different ploidy for identical chromosomes. On the other hand, there is a significant difference in chromosome aneuploidy (unpaired two samples student's t-test, p = 0.028) when SE parasites, patient and clones, were compared to NE parasites, patient and clones.

Cluster analysis based on chromosome copy number provides insight on three levels: first, that similarities and differences exist between all parasites examined; second, validation that clones derived from the same patient strain are highly similar; and third, that differences in chromosome copy are not correlated with the parasite geographic origin. Similar to earlier studies [16, 19, 20, 25] where aneuploidy was examined in clinical isolates of *Leishmania* from India and Nepal (*L*. *donovani*), in sand flies from Turkey (*L*. *infantum*), and in laboratory strains (*L*. *major*, *L*. *braziliensis*, *L*. *donovani*, *L*. *infantum*, *L*. *mexicana* and *L*. *panamensis*); all the Ethiopian *L*. *donovani* lines examined showed aneuploidy, and each has a different karyotype. Seven chromosomes (17, 25, 27, 30, 32, 34 and 36) were disomic in all lines examined, as was chromosome 3 with the exception of one strain AM554. Four of these chromosomes (30, 32, 34 and 36) were used for normalization of average chromosome read coverage as described by Rodgers et al [18]. Interestingly, disomy was also observed for five of these chromosomes, 17, 25, 30, 34 and 36, in all the 17 Indian and Nepalese *L*. *donovani* lines originally studied [16], and in almost all 206 strains from the Indian subcontinent recently examined [20]. Several other chromosomes (18, 19, 21 and 28) that were disomic in all the Indian and Nepalese lines showed somewhat more diverse ploidy (2 to 3 copies) in the Ethiopian lines. Chromosome 31 was polysomic in all the lines used in this study. Even GR363sk/cl.I and GR363sk/cl.II, which showed the least aneuploidy of all the lines examined, were still trisomic for chromosome 31. Chromosome 31 is polysomic in every Old World *Leishmania* strain examined to date [16, 19, 25].

**Fig 2:**
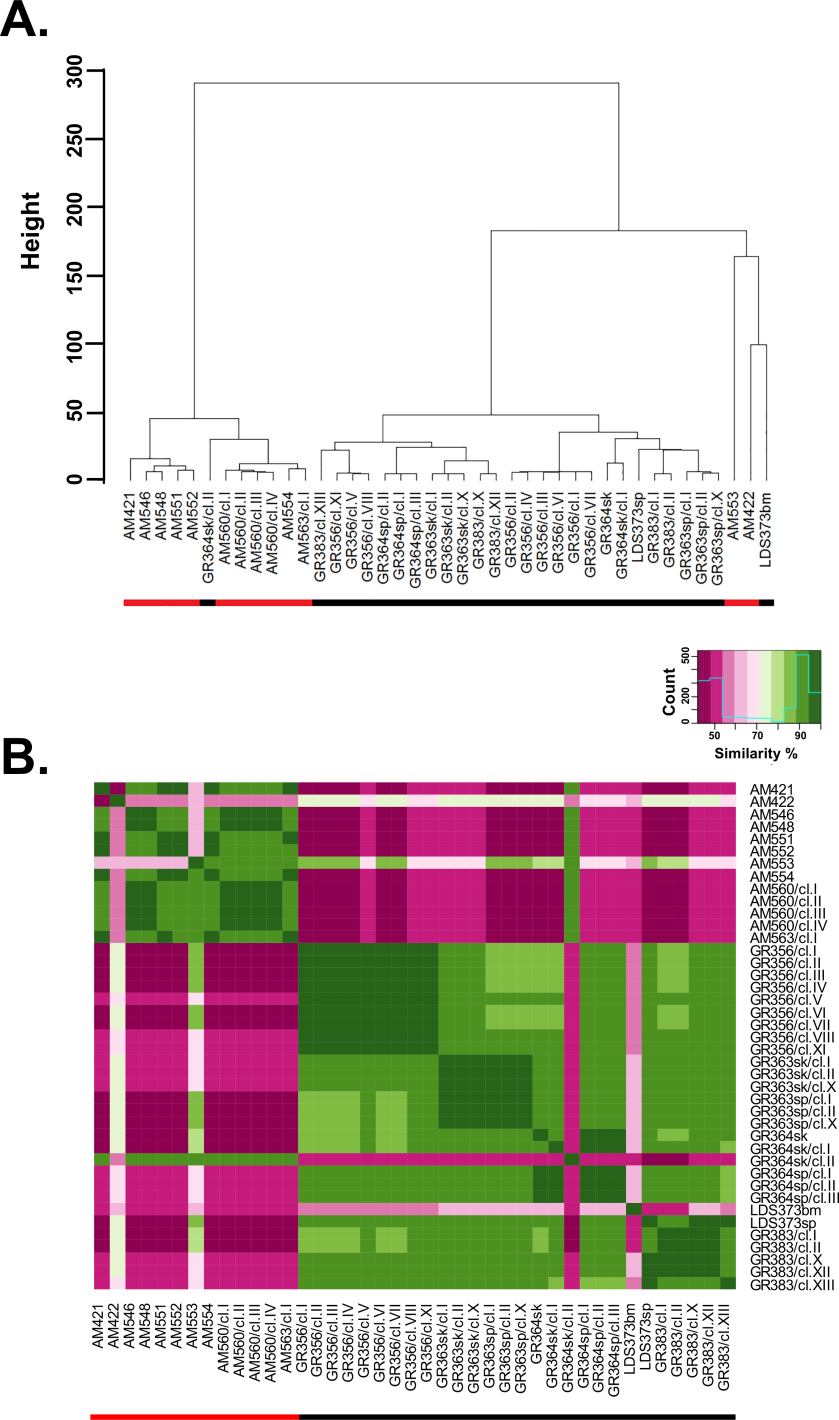
Phylogentic tree and similarity analysis based on SNP. Panel A. Cluster analysis based on Euclidian distance between SNP similarity profiles among all 41 NE (black bar under name) and SE (red bar under name) lines. The resulting phylogenetic tree describes the genetic diversity among Ethiopia isolates within the two main *Leishmania* populations. B. Comparison between SNP profiles of *Leishmania donovani* strains and clones from northern and southern Ethiopia. Heat map allows visualization of similarities between all SNPs from SE and NE strains and clones. Each colored square in the matrix indicates the percent SNP similarity for a strain/clone listed on the right compared to strain/clone listed along the bottom of the matrix. The heat map was generated with R packages; *stats* and *gplots*. The green range indicates on identity level ≥ 70% and the pink < 70%. Darker green/pink point to higher or lower identity, respectively.

**Fig 3:**
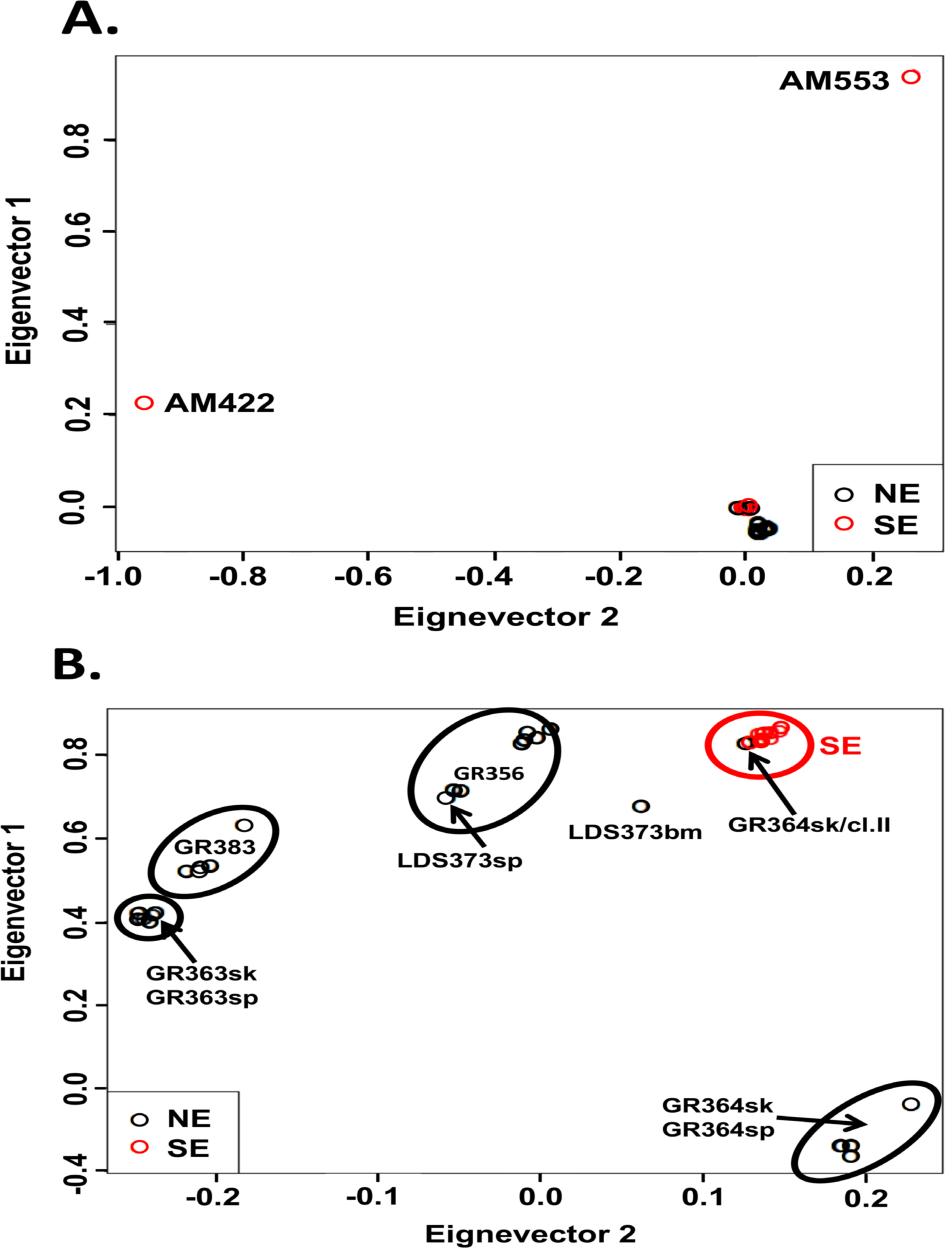
Principal component analysis (PCA) based on SNP of *Leishmania donovani* NE and SE lines. PCA analysis was performed after pruning SNPs showing high linkage disequilibrium (LD). SNPs with LD>0.9 are not included. Panel A. PCA of all 41 NE and SE lines. The two atypical SE parasites, AM422 and AM553, fall in eigenvectors 1 and 2 relatively far from both populations (EV1 = 0.229 EV2 = -0.954 and EV1 = 0.945 EV2 = 0.265, respectively). Two NE parasites, GR364sk/cl.II and the atypical LDS373bm, fall with the SE population. Despite the short distance between typical SE (red circles) and NE (black circles) population, these two populations are physically well defined. Panel B. PCA of 39 NE and SE lines without atypical SE strains. The typical SE population (red circles) is very homogenous and falls in a tight cluster unlike the NE population, which is very heterogeneous but still distinct from the SE population. LDS373bm falls equidistance between the SE population and LDS373sp both isolated from the same patient. Line GR364sk/cl.II still clusters together with the SE population (black circle on the middle of the SE cluster). Interestingly lines from spleen and skin taken from the same patients (GR363sp and sk; and GR364 sp and sk) are clustered together and create a well-defined population.

Chromosome copy number was compared using clones from several strains (AM560, n = 4; GR356, n = 9; GR363sp, n = 3; GR363sk, n = 3; GR364sp, n = 3; GR383, n = 5). In one case (GR364sk, n = 3) two clones and the original patient strain were examined. Validation of aneuploidy similarities was carried out using clustering based on ploidy patterns taking into account all 36 chromosomes. What is readily apparent, from both the heat map and dendrogram (Fig 1), that in most cases karyotypes of clones isolated from the same strain are highly similar. For instance, the karyotypes of the five GR383 clones (I, II, X, XII and XIII) are almost identical (unpaired student's t-test, p > 0.73), and fall in a tight cluster. This is also seen for all the clones of GR363sp, GR363sk, GR364sk and AM560, and 8/9 clones of GR356. Only two clones, GR356/cl.I and GR364sk/cl.II, show karyotypes significantly different from their sister lines or the patient strain from which they were derived, and fail to cluster with the former (Fig 1). Interestingly, clone GR356/cl.1 groups with its sister lines based on SNP analysis (Figs 2 and 3), and exhibits a k26-PCR amplicon identical to the other NE strains (290 bp). Of note, clone GR364sk/cl.II had a k26-PCR amplicon (450 bp) similar in size to SE strains, even though it was derived from GR364sk, a NE strain with a 290 bp amplicon typical of the NE region [7]. This strain was isolated from a HIV-VL patient, and the sister clone, GR364sk/cl.I which is very similar to the patient strain also has a k26—PCR product of 290 bp. Clone GR364sk/cl.II also groups with the SE strains by SNP analysis (Figs 2 and 3), but is distinct from them suggesting that this patient might have had a mixed infection.

Cluster analysis of the karyotype data doesn’t separate the SE and NE strains by geographic region instead they are mixed together, interspersed among each other. Despite this, some differences in chromosome copy number between the two populations are apparent. Chromosomes 13, 15, 16, 23, 28, and 31 show significantly higher average ploidy for SE strains compared to NE strains, while chromosome 4 is the only chromosome where the NE strains show a significantly higher average ploidy than the SE population (Table 1 and S2 Table). In addition, we found that SE strains show a significantly higher average total chromosome somy (2.52 ± 0.09) than the NE strains, (2.35 ± 0.06; paired t-test, p = 0.0016), perhaps indicating that the SE strains tend towards higher polyploidy than the NE strains (S2 Table).

**Table 1:** Average chromosome ploidy as a separator of the two Ethiopian parasite populations*.

Chromosome	SE_mean_	NE_mean_	SE_mean_/ NE_mean_	p-value
4	2.13	2.63	0.81	0.041
13	3.19	2.44	1.31	0.002
15	2.68	2.08	1.29	0.038
16	2.62	2.14	1.22	0.02
23	3.94	2.82	1.40	0.018
28	2.32	2.01	1.15	0.034
31	4.36	3.66	1.19	0.002

*Where multiple clones exist for an individual isolate, the value used in calculating average chromosome ploidy is the median ploidy of the clones for that strain.

Finally, parasites from different organs of three HIV-VL co-infected patients were examined. Parasites isolated from the skin or spleen of the same patient form separate groups, and the karyotypes of parasites isolated from the respective sites show greater differences, in most cases, than clones generated from the same site, either skin or spleen. GR364 spleen and skin strains, with the exception of GR364sk/cl.II, form separate clusters (Fig 1). Overall these five lines (skin–original patient strain and clone I, versus spleen—all three clones) show no significant difference in chromosome ploidy distribution, i.e. karyotype. However, when each GR364 spleen and skin chromosome was compared on an individual basis, 6 out of 36 chromosomes (Ld4, 5, 8, 14, 20 and 31) show significant differences in ploidy (p = 0.0004, 0.04, 0.01, 0.05, 0.01 and 0.001, respectively). This probably accounts for the fact that the skin and spleen strains form separate subgroups (Fig 1). Likewise, GR363sk and GR363sp taken from the skin and spleen, respectively, of the same patient cluster in separate branches of the dendrogram (Fig 1). Significant overall differences in ploidy between the GR363 skin and the spleen clones (two tailed paired t-test, p = 0.0067) was observed. Interestingly, the spleen clones from this patient demonstrate on average higher ploidy than the skin clones. Finally, strains isolated from the spleen and bone marrow of one patient, LDS373sp and LDS373bm respectively, also show different karyotypes (7/36 chromosomes differ), even though they cluster together on the dendrogram. These parasites also show different k26—PCR fragment sizes, gene CNV and SNP profiles. An overall difference in ploidy was found in chromosomes 1, 4, 6, 20 and 35 by the comparison of all spleen against skin clones. The differences in chromosome ploidy of strains isolated from different organs may result from clonal selection of the parasites in the host due to specific selective pressures at the infection site.

### Single nucleotide polymorphism analysis

SNP calling, compared to the *L*. *donovani* reference strain (BPK282A1), for each of the strains and clones was carried out as described in material and methods. The two parasite populations, SE and NE, show significantly different number of SNPs on average, ~153K and ~168K (p < 0.043) respectively, compared to the Indian reference strain (R). In addition, the percentage of homozygous and heterozygous SNPs within each geographic population (S3 Table), represented by a single alternate allele (A), show significant differences i.e., for the SE (mean homozygous AA = 87.2% and heterozygous RA = 12.7%, respectively, p<0.05) and the NE populations (mean homozygous AA = 83.3% and heterozygous RA = 16.6%, respectively, p<0.05). The relationship between SE and NE strains and clones based on whole genome SNP pair-wise analysis is shown in Fig 2. Each colored square in the matrix indicates the percent SNP similarity for a strain/clone listed on the left compared to strain/clone listed along the bottom of the matrix. Unlike the chromosomal aneuploidy profiles (Fig 1), SNP population analysis divides the *L*. *donovani* population into large clades or groups based on geographical distribution (Fig 2). This is easily seen both in the hierarchical cluster tree where SE and NE parasites each form separate groups with clones from each strain showing highest similarity to each other (Fig 2A) and the heat map (Fig 2B). The only exceptions are three atypical strains/clones (AM422, AM553, and LDS373bm) which fall outside the main clades, and clone GR364sk/cl.II, as mentioned above, which groups with the SE rather than the NE parasites. The two atypical SE strains; AM422 and AM553 fall closest to the NE clade, yet are distinct from the NE strains. Interestingly, LDS373bm does not cluster with the spleen strain, LDS373sp, isolated from the same patient, even though the karyotypes are similar. AM422, AM553 and LDS373bm seem to have SNP profiles falling between the NE and SE populations.

Principal component analysis on the SNPs was also used to examine the population structure. SNPs showing high linkage disequilibrium were removed prior to analysis by SNP pruning [26] (S4 Table). As can be seen in Fig 3A, two clusters or near-clades containing most the NE (black circles) or SE (red circles) strains and clones are observed. The two atypical SE strains, AM422 and AM553, are outliers falling far outside both clusters (EV1 = 0.229, EV2 = -0.954 and EV1 = 0.945 EV2 = 0.265 respectively), while the two atypical NE strains, GR364sk/cl.II and LDS373bm, group together with the rest of the SE population confirming the results shown in Fig 2. PCA was repeated excluding the two atypical SE strains to allow better separation of the remaining 39 isolates (Fig 3B). All the SE strains still group together in one dense cluster that includes GR364sk/cl.II suggesting that these strains are more homogenous, however the NE strains show more diverse distribution with each strain and its clones forming a separate cluster. LDS373bm no longer groups with the SE strains.

SNPs in protein coding regions were examined, and SNPs unique to the SE and/or NE populations identified. No significant difference in the percentage of synonymous, nonsynonymous or nonsense mutations was found between SE and NE parasite populations: 47%, 52.6% and 0.4% versus 50.0%, 49.8% and 0.2%, respectively. Altogether 683 common genes containing at least one SNP causing either a nonsynonymous or a nonsense mutation are present in both NE and SE parasites. The remaining SNPs resulting in nonsynonymous or nonsense mutations are only found in either SE (412 genes) or NE (595 genes) parasite populations (S5 Table). As such, SNPs in these genes are unique markers for parasites in each geographic region.

**Fig 4:**
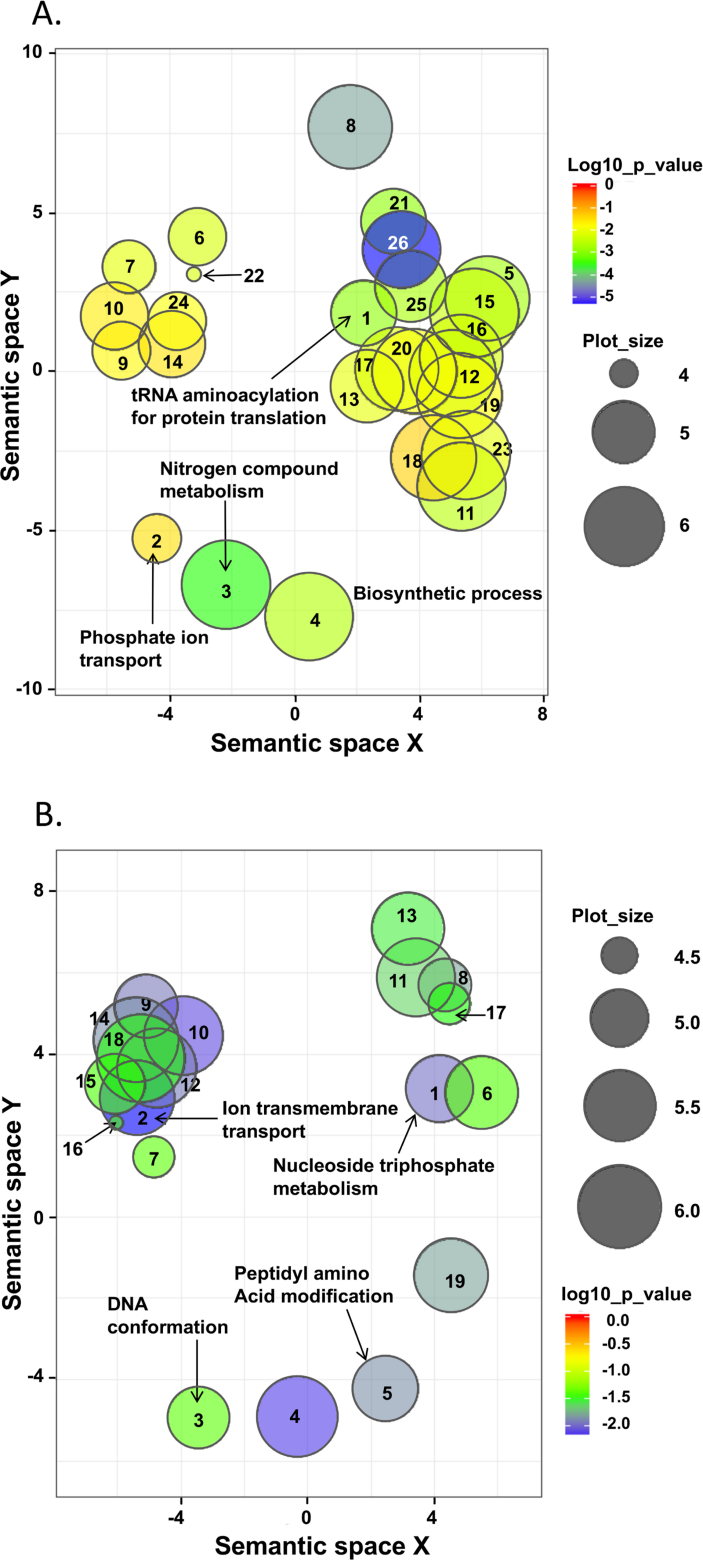
Scatterplots after reduction of semantic redundancy in enriched Gene Ontology (GO) terms for unique SNP containing genes of northern and southern *Leishmania donovani* populations. Removal of redundant GO terms was carried out using the web program REVIGO with C set at 0.7 [27]. Enriched GO terms are graphed in two-dimensional semantic space with terms that semantically similar closer together. The semantic space units have no intrinsic meaning. Enrichment p-values are shown by circle color as indicated in the key to the right of each panel, while circle diameter indicates the frequency of the GO term i.e. general terms are larger. Numbers in each circle refer to the GO terms listed in S7 Table. A. Northern Ethiopia (NE). Panel B. Southern Ethiopia (SE).

Gene Ontology enrichment analysis of the “unique” SNP containing genes found in each population indicates that the proteins are involved in different biological processes (S6 Table). A web based semantic cluster algorithm, REVIGO, was used to remove redundant GO terms [27]. After removal of redundant GO terms the remaining terms were graphed as scatterplots in two-dimensional space according to semantic similarity. Semantically similar GO terms should remain close together in the plot, and size of the circle indicates the frequency of GO term. Unique SNPs in NE parasites (Fig 4A and S7 Table) are associated with biological processes such as tRNA aminoacylation for protein translation, glutamine family metabolism, regulation of transferase activity e.g. protein kinases, and phosphate ion transport, while those in SE parasites are primarily associated with cation transmembrane transport, purine nucleoside triphosphate and nucleobase metabolism and DNA conformation (Fig 4B). Similar differences are also noted when the molecular functions of the genes with unique SNPs are analyzed. Unique SNPs in the NE population are concentrated mainly in genes involved in glutamine family biosynthesis and metabolism, tRNA aminoacetylation, pyrimidine metabolism, and cyclins involved in protein kinase regulation during cell division. On the other hand, unique SNPs associated with the SE population are found in genes such as glutathione metabolism, protein translation initiation and elongation factors, transport and oxidoreductase activity (S5–S7 Tables).

Several genes associated with the development of leishmanial drug resistance also contain nonsynonymous SNPs and/or nonsense mutations. A unique SNP, only present in the NE population, was identified in the aquaglyceroporin *(LdBPK_310030)* gene, a protein that plays a role in trivalent antimony (Sb^III^) uptake, located on chromosome 31 [20]. This unique heterozygote nonsynonymous mutation g.7444A>T causes an amino acid exchange (Ser251Thr) in TML-5 of aquaglyceroporin, and is only found in the NE population (S5 Table). The MRPA gene (PGPA) encodes an ABC-thiol transporter (*LdBPK_230290*.*1)* that sequesters thiol-Sb conjugates and is also involved in antimony resistance [28]. This gene contains several unique nonsynonymous SNPs unique to the NE (four homozygote and one heterozygote), and SE (two homozygote) populations (S5 Table). It is not clear how these unique SNPs affect transporter function, as no difference in response to antimonial chemotherapy between *L*. *donovani* isolates from NE and SE has been reported.

### Gene copy number variation

Comparative read coverage was examined for 41 SE and NE isolates using a sliding window (5000 bp) in order to detect genomic copy number variation (CNV) as described in Material and Methods. Chromosome somy was not taken into account at this stage. While both increases, and decreases in CN were observed (S8 Table), increases in CN (>2) were more prevalent occurring 83% of the time. In addition, a significant overall difference (p < 0.00001) in average CNV between the NE and SE populations was noted (Table 2). Sixty-two different genes showed significant differences in CN between the SE and NE populations (S9 Table and S1 Fig). In the SE strains, genes with an average CN < 1.5 are primarily found on chromosomes 10, 11, 12, 22, 27, 31, 34, and 36; and include the folate-biopterin transporters, ABC transporters, ATP-binding cassette protein, D-lactate dehydrogenase, branched-chain amino acid aminotransferase, amastin-like proteins, phosphoglycerate mutase, tartrate-sensitive acid phosphatase, mitogen activated protein kinase homolog, as well as numerous uncharacterized proteins. Genomic CNV of the atypical NE strains was very similar to that observed for the SE strains. On the other hand, only one gene, an amastin-like protein (*LdBPK_341700*) on chromosome 34, shows an average CN <1.5 in a majority of NE strains. Interestingly, low copy number genes were more prevalent in the SE population with 84% of all strains and clones exhibiting an average CN < 1.5 over all genes.

**Table 2:** Summary of copy number variation (CNV) in each *L*. *donovani* strain*.

WHO Code	No. complete gene deletions (CNV = 0)	No. haploid genes (CNV = 1)	No. polyploidy genes (CNV>2)	Average polyploidy	Average CNV
MHOM/ET/2009/AM421	0	19	19	3.31	2.16
MHOM/ET/2009/AM422	0	7	19	3.42	2.77
MHOM/ET/2010/AM546	0	18	20	3.15	2.13
MHOM/ET/2010/AM548	0	18	18	3.16	2.08
MHOM/ET/2010/AM551	0	17	25	3.12	2.26
MHOM/ET/2010/AM552	0	16	21	3.14	2.21
MHOM/ET/2010/AM553	0	4	24	3	2.71
MHOM/ET/2010/AM554	0	16	17	3.17	2.12
MHOM/ET/2010/AM560^#^	0	14	44	3.16	2.65
MHOM/ET/2010/AM563	0	19	53	3.3	2.71
MHOM/ET/2009/GR356^#^	0	2	31	3.1	3.01
MHOM/ET/2010/GR363sp^#^	0	4	5	3.8	2.62
MHOM/ET/2010/GR363sk^#^	0	3	5	3.8	2.74
MHOM/ET/2010/GR364sp^#^	0	4	22	3.32	2.96
MHOM/ET/2010/GR364sk^#^	0	7	34	3.31	2.95
MHOM/ET2010/GR379	0	3	16	3.25	2.90
MHOM/ET/2010/GR383^#^	0	5	74	4.51	4.23
MHOM/ET/2009/LDS373bm	2	10	158	3.04	2.88
MHOM/ET/2009/LDS373sp	0	2	18	3.38	3.15
MHOM/SD/1998/S3570	0	4	27	3.77	3.42

*A summary of the absolute number of different genes showing CN≠2 over all isolates examined in this study e.g., complete gene deletion (CN = 0), deletion of one copy (CN = 1) or greater than two copies of a gene (CN>2). Isolates from southern Ethiopia (SE) have codes beginning with AMxxx, where xxx represents the strain number. Isolates from northern Ethiopia (NE) have codes beginning with either GRxxx or LDSxxx, where xxx represents the strain number.

#Multiple clones exist for the same patient strain, and the value given represents the average number of genes showing gene copy number different from CN = 2.

One striking difference between the NE and SE strains and clones is in the number of folate/biopterin transporter (FBT) gene(s) on chromosome 10 (Table 3, S10 Table and S2 Fig). *Leishmania* are auxotrophs for folic acid, and 14 different members of FBT family have been identified in *L*. *infantum* [29]. Several of these genes are known to play roles in parasite drug resistance and viability. Eight of the 13 *L*. *donovani* FBT homologs are located on chromosome 10, of which 7/8 are present in a tandem array. This chromosome is disomic in most leishmanial strains examined to date [16, 18–20, 25]. Interestingly, three of the FBT genes present in this tandem array on chromosome 10 (*LdBPK_100390*, *LdBPK_100400* and *LdBPK_100410*) show gene amplification in several NE (GR364sk, GR364sp and GR356) and Sudanese parasites (S10 Table). In addition, *LdBPK_355160* on chromosome 35, an ortholog of *L*. *infantum* biopterin transporter 1, also a member of the FBT family, is amplified in the NE strain GR383 (CN = 5), but not the other NE isolates.

**Table 3:** Haploid gene copy number variation in *leishmania donovani* from Northern and Southern Ethiopia*.

Gene Id	Annotation**	Southern Ethiopia			Northern Ethiopia		
		CN^§^	Chr ploidy	CN	CN^§^	Chr ploidy	CN
LdBPK _080780; LdBPK _080790	Amastin-like protein	2.3	2.9	**3.3**	1.7	2.5	**2.2**
LdBPK _100390	Folate/biopterin transporter	1.2	2.1	**1.3**	2.7	2.0	**2.8**
LdBPK _100400; LdBPK _100410	Folate/biopterin transporter	1.0	2.1	**1.0**	2.7	2.0	**2.8**
LdBPK _191340; LdBPK _191350	Glycerol uptake protein	2.9	2.0	**3.0**	2	2.3	**2.3**
LdBPK _231050	Quinone oxidoreductase	3	4.5	**6.7**	2.0	3.0	**3.0**
LdBPK _231240	Hydrophilic acylated surface protein	2.8	4.5	**6.2**	2.0	3.0	**3.0**
LdBPK_271940	D-lactate dehydrogenase	1.1	2.0	**1.1**	2.0	2.0	**2.0**
LdBPK_271950	Branched-chain amino acid aminotransferase	1.1	2.0	**1.1**	2.0	2.0	**2.0**
LdBPK_282040	PUC	2.9	2.2	**3.2**	1.8	2.0	**1.8**
LdBPK _282050	Zinc transporter	2.9	2.2	**3.2**	1.8	2.0	**1.8**
LdBPK _291620; LdBPK _291630	Major facilitator superfamily	1.7	2.5	**2.1**	3.0	2.1	**3.2**
LdBPK _301630	Ferric reductase	2.3	2.0	**2.3**	3.2	2.0	**3.2**
LdBPK _303210	Mitosatin	3.5	2.0	**3.5**	2.0	2.0	**2.0**
LdBPK _303220	RNA binding protein	3.5	2.0	**3.5**	2.0	2.0	**2.0**
LdBPK _303230	3-hydroxy-3-methylglutaryl-CoA reductase	3.5	2.0	**3.5**	2.0	2.0	**2.0**
LdBPK _311470	PUC	1.1	4.4	**2.5**	2.0	3.8	**3.8**
LdBPK _311630	PUC	2.9	4.3	**6.4**	2.0	3.8	**3.8**
LdBPK _311640	Diphthine synthase-like protein	2.9	4.4	**6.4**	2.0	3.8	**3.8**
LdBPK _311650	PUC, DUF84	2.9	4.4	**6.4**	2.0	3.8	**3.8**
LdBPK _311660	3-ketoacyl-coa thiolase	2.9	4.4	**6.4**	2.0	3.8	**3.8**
LdBPK _311920	Mannosyltransferase	2.5	4.4	**5.6**	2.0	3.8	**3.8**
LdBPK _311930	Ubiquitin-fusion protein	2.5	4.4	**5.6**	2.0	3.8	**3.8**
LdBPK _341700	Amastin-like protein	2.5	2.0	**2.5**	1.4	2.0	**1.4**
LdBPK _342650; LdBPK_342660	Amastin-like protein	1.2	2.0	**1.2**	2.5	2.0	**2.5**
LdBPK _366740	Tartrate-sensitive acid phosphatase	1.2	2.0	**1.2**	2.0	2.0	**2.0**
LdBPK _366750	PUC	1.2	2.0	**1.2**	2.0	2.0	**2.0**
LdBPK _366760	Mitogen activated protein kinase	1.1	2.0	**1.2**	2.0	2.1	**2.0**

*≥ 60% of the clones and strains show variation from diploid.

**PUC–putative uncharacterized proteins.

§ Average gene copy number–CN; Average chromosome ploidy–Chr ploidy.

SE parasites exhibit the opposite trend for these three genes (*LdBPK_100390*, *LdBPK_100400* and *LdBPK_100410)* on chromosome 10 showing loss of heterozygosity in eight, ten, and ten out of eleven SE strains, respectively (S10 Table). Loss of heterozygosity was also observed for one additional FBT gene on chromosome 10 (LdBPK_100380) in the SE strain AM553. Interestingly, two atypical NE strains, LDS373bm and GR364sk/cl.II, which group with the SE strains by SNP analysis also show loss of heterozygosity for the same three FBT genes on chromosome 10. The average haploid CN taking into account chromosome somy for these three genes is 2.75 in the NE strains versus 1.05, 1.25 and 1.25 respectively in the SE strains (Table 3).

**Fig 5:**
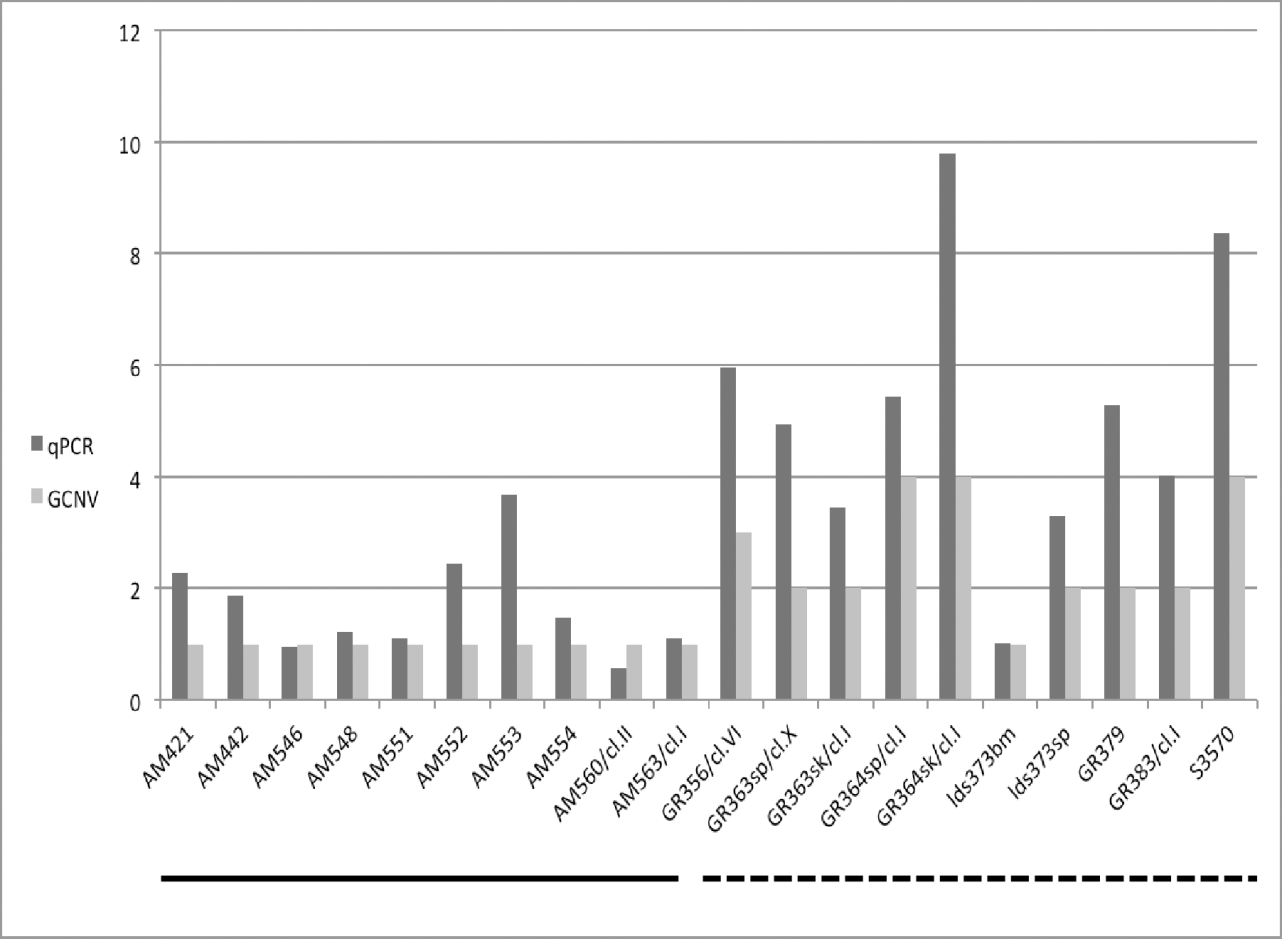
Comparison of folate transporter 1 (LdBPK_100400.1) genomic DNA copy number in northern and southern Ethiopian *Leishmania donovani* strains by quantitative polymerase chain reaction (qPCR) and cn.mops (GCNV). *L*. *donovani* strains and clones are shown on the x-axis, while estimated FT1 copy number is given on the y-axis. SE strains (n = 9, line under x-axis), and NE (n = 9) and Sudanese (S3570) strains (dashed line under x-axis).

Genomic CN for *LdBPK_100400* (*L*. *infantum* FT1 homologue) in the NE and SE leishmanial strains was also determined by qPCR (Fig 5) in 20 strains and clones using a novel dual priming oligonucleotide system [30]. qPCR tended to give higher CNs for this gene than found by computational analysis (cn.mops), however there was a good correlation overall between FT1 CN based on computational estimation with cn.mops [31] and qPCR (ρ = 0.91). Strain LDS373bm also showed loss of heterozygosity by qPCR, similar to what was found above by computational analysis.

In addition, both methods show significant difference in FT1 CN between the SE and NE populations. The mean FT1 CN for the two different methods and populations is as follows: qPCR; SE_mean_ = 0.79, NE_mean_ = 2.44, p = 0.00034; cn.mops; SE_mean_ = 1, NE_mean_ = 2.6, p = 0.00009 confirming the trend toward loss of heterozygosity in the SE and amplification in the NE strains / clones examined.

**Fig 6:**
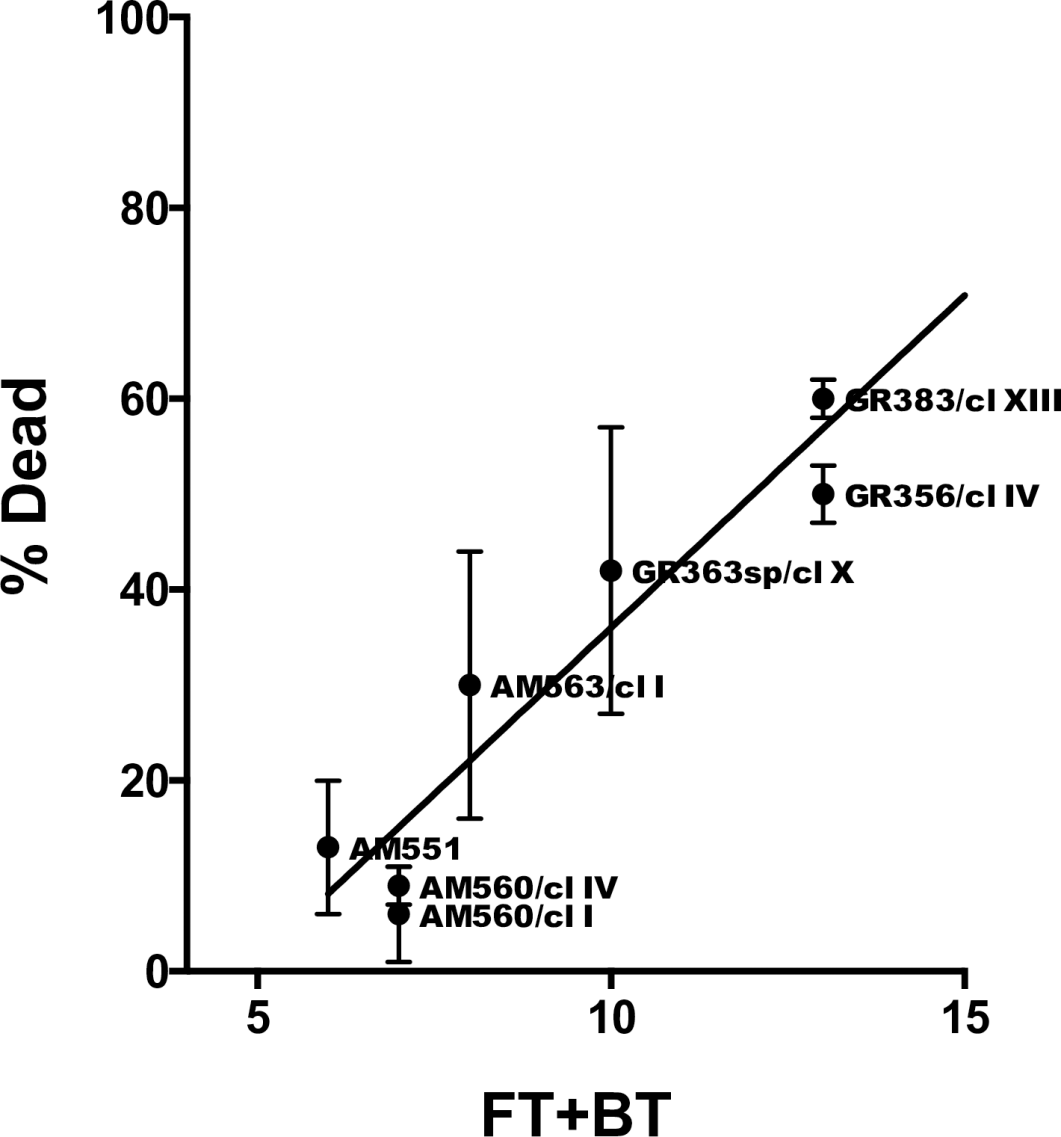
Folate transporter gene copy number is correlated with greater sensitivity to methotrexate. Effect of Methotrexate on parasite growth (%Dead) was plotted as a function of the total gene copy number of folate (LdBPK_100380; LdBPK_100390; LdBPK_100400 and LdBPK_100410) and biopterin transporters (LdBPK_355160) showing variation on chromosomes 10 and 35, respectively (FT+BT). CN was predicted by cn.mops. %Dead—percentage parasites killed following incubation with 0.5mg/ml methotrexate as described in Material and Methods. Average of n = 3 independent experiments ± s.e. Isolates from southern Ethiopia (SE) have codes beginning with AM. Isolates from northern Ethiopia (NE) have codes beginning with GR.

FT1 is the main transporter for folate. Resistance to methotrexate (MTX) is correlated with reduced folate uptake [32, 33], and CN for this gene was reduced in some resistant parasites [29, 34]. Therefore, the effect of MTX on the viability of eight SE and NE strains/clones that vary in FT1 CN was examined (Fig 6). Several of the SE and NE strains examined also show CNV for other FBT genes on chromosome 10 flanking FT1 (S10 Table). All of the SE strains/clones tested are single copy for FT1 and are significantly less sensitive (6–30% growth inhibition) to MTX (0.5 mg/ml) than the NE strains/clones (42–78% growth inhibition), p = 0.005; and a linear correlation (r^2^ = 0.937) between CN, for the genes demonstrating CNV on chromosomes 10 (*LdBPK_100380*, *LdBPK_100390*, *LdBPK_100400* and *LdBPK_100410)* and 35 (*LdBPK_355160)*, and sensitivity to MTX was observed (Fig 6 and S11 Table). NE parasites are significantly more sensitive (p = 0.02 to 0.0009) to inhibition by MTX over a wide range of concentrations (33 to 900 μg/ml) when grown at limiting folate concentrations (S3 Fig). A high correlation between FT1 CN and sensitivity to MTX was found (Pearson's correlation coefficient ρ = 0.85, p = 0.007).

Plasticity in gene organization has been reported for several *Leishmania* species with the number of gene copies varying between isolates from the same species [35–37] and changes in gene dosage may be correlated with differences in protein expression [18]. For instance, the region on chromosome 10 containing *LdBPK_100480*, *LdBPK_100510*, *LdBPK_100520* and *LdBPK_100521* encodes a Zn-binding protein whose function is unknown, two tandem copies of gp63 and an uncharacterized protein, respectively (S8 and S9 Tables). This region is amplified (CN = 3) in 3/10 SE strains and one SE-like NE clone, GR364sk/cl.II that clusters by SNP analysis with the SE strains. All other strains are diploid for this region. Gp63 is a protease involved in parasite virulence and survival [38, 39], and is frequently present on chromosome 10 in other species as a multicopy gene family e.g., *L*. *infantum* (*LinJ*.*10*.*0490*, *10*.*0500*, *10*.*0510*, *10*.*0520* and *10*.*0530*) or *L*. *major* (*LmjF*. *10*.*0460*, *10*.*0465*, *10*.*0470* and *10*.*0480*). Likewise, on chromosome 19 there are two glycerol uptake proteins (*LdBPK_191340* and *LdBPK_191350*) that have an additional gene copy (CN = 3) in 9/10 SE strains and the SE-like clone (GR364sk/cl.II). Interestingly, in other species these genes are part of a tandem multicopy gene family (*L*. *infantum* 7 genes, *L*. *major* 6 genes, *L*. *braziliensis* 8 genes) that may be involved in the remodeling of lipids on glycerol phosphoinositol lipid anchors. Amplification of the 48 kb H-region on chromosome 23 has been associated with drug resistance *in vitro* [40, 41]. Part of this region is also amplified in some wild-type strains [40, 41]. Deletions (CN = 1) or duplications (CN = 4) of part of the H-region (9 kb) were seen in several SE and NE parasites, respectively. The deleted region was found in 3/10 SE strains and contains the genes coding for the ABC-thiol transporter (MRPA) (*LdBPK_230290*), involved in resistance to antimony [42], and argininosuccinate synthase (*LdBPK_230300*), involved in arginine synthesis [43]. Interestingly a similar region is amplified in 8 clones from 2 different NE patient strains, and contains the genes coding for argininosuccinate synthase (*LdBPK_230300*), a putative uncharacterized protein (*LdBPK_230270*), the Terbinafine resistance locus protein (Yip1) (*LdBPK_230280*), and the PTR1 gene (*LdBPK_230310*). These genes are present in the H-region and frequently amplified in some drug resistant cell lines [44], however they were unchanged, diploid, in all the other parasites belonging to NE and SE populations. This is similar to CN analysis of antimony resistant and sensitive *L*. *donovani* strains from Nepal where amplification was not observed for the H-region genes [16], even though MRPA, and in some cases the PTR1 gene, were shown to be amplified in naturally resistant parasites examined by other techniques [45, 46]. Finally, CNV was also found in part of a 15.8 kb region located on chromosome 36 known as the MAPK locus (*LdBPK_366740*, *LdBPK_366750*, *LdBPK_366760* and *LdBPK_366770*). Amplification of this region was found in antimony resistant *L*. *donovani* from Nepal and associated with higher gene dosage in drug resistant lines [16] [47]. Interestingly, CN of 3/4 genes found in this locus were significantly lower (p ≤ 0.5 x 10^−7^) in the SE and SE-like NE strains/clones than the NE strains/clones (S8 and S12 Tables). No significant different in CNV between the NE and SE parasites, CN = 2 in 40/41 strains and clones, was observed in the case of the histidine secretory acid phosphatase (*LDBPK_366770*) which is considered part of the MAPK-locus and amplified in antimony resistant parasites [16]. The only exception was seen with the SE-like NE strain (LDS373bm) that show a complete deletion of *LDBPK_366770*, as well as *LDBPK_366780* (CN = 0).

When haploid gene CN is calculated, taking into account both gene CNV and chromosome ploidy, most of the differences between the NE and SE parasite populations (Table 3 and S13 Table) still remain even though chromosome polyploidy is statistically more common in SE parasites.

## Discussion

Genome wide sequencing (WGS) and analysis of pathogens has proven widely useful for investigations on molecular epidemiology and evolution; genotype—phenotype associations; identification of genes involved in various biological processes such as drug resistance and virulence; as well as new targets for drug and vaccine development [16–18, 21, 48, 49]. Developments in next generation sequencing over the last two decades have provided a relatively low cost, fast pipeline for the exploration and comparison of *Leishmania* genomes. This study focused on comparison and analysis of WGS data from a large number of *L*. *donovani* strains and clones (n = 41) originating from fifteen VL patients in southern and northern Ethiopia. Previous studies on population genetics using multilocus microsatellite typing (MLMT) or individual gene sequences suggest that *L*. *donovani* is comprised of distinct populations associated with specific geographic regions in East Africa [7, 15, 50], and that African parasites are in large part distinct from those found on the Indian subcontinent [13, 17]. Differences have also been documented in the parasites, sand fly vectors and host responses between these geographic regions e.g., sensitivity of antigen based serodiagnostic assays [51], clinical response to paromomycin [6], incidence of PKDL [52, 53]. Our whole-genome sequence data confirms the presence of two very different populations of *L*. *donovani* in the region, exemplified by an absolute difference of ~15,000 SNPs between the NE and SE populations. These parasite populations likely arose due to unique evolutionary pressures associated with local sand fly species, hosts, reservoirs, ecology, and other factors. A unique advantage of whole-genome data is that it gives us a comprehensive catalog of genetic variation that could underpin these adaptations.

When chromosome aneuploidy is analyzed, a picture appears suggesting great diversity among the Ethiopian strains within each population. This picture is unlike that shown for the Ethiopian reference strain LV9 where all 35 chromosomes, except for chromosome 31, were disomic [18]. This reference strain was extensively passaged in numerous laboratories since first isolated from a VL patient in 1967. Unlike the reference strain, all the isolates used in this study were rapidly cryopreserved and only briefly cultured under identical conditions prior to DNA purification for WGS, yet they still show unique, highly variable karyotypes compared to strains from the same geographic population or strains isolated from different organs of an identical patient. This indicates that chromosome aneuploidy, unlike SNPs, cannot be used to map leishmanial population genetics. Interestingly, MLMT analysis of *L*. *donovani* strains from Libo Kemkem, a previously non-endemic region in NE where an outbreak of VL occurred in 2004–2005, also demonstrated high genetic diversity among parasites isolated from patients, including a unique genetic group that shared several alleles with strains from SE [50].

*Leishmania* chromosome ploidy in individual cells can change rapidly in response to environmental conditions, even routine culture, resulting in mosaic aneuploidy [54], however in this study individual clones generated from patient strains generally showed similar karyotypes clustering together, and when examined, were highly similar to the parental patient isolate. These results suggest that the average karyotype for each strain is relatively stable i.e., minimally affected by culturing and/or cloning prior to DNA extraction. Downing et al [16] also reported that chromosome aneuploidy was stable in culture for 17 Nepalese *L*. *donovani* strains even though they also showed diverse karyotypes. This suggests that the karyotypes observed for the parasites in this study are probably very similar to the original patient isolate, assuming changes don’t take place upon differentiation from intracellular amastigote to extracellular promastigote. However, this can only be confirmed by direct measurement of aneuploidy in parasites taken directly from patients without prior culturing, something not currently possible.

Similar to previous reports, chromosome 31 was supernumerary in all 41 Ethiopian strains and clones studied. This appears to be a defining characteristic in all *Leishmania* species examined so far [16, 18, 19, 25, 55]. As genes involved in iron metabolism and related functions are highly enriched on chromosome 31, it has been suggested the chromosome polyploidy arose to expedite iron uptake, and that expression of Iron–Sulfur proteins that are important in oxidation-reduction reactions, and synthesis of metabolites essential for parasite survival and growth [55].

One interesting finding is that strains concurrently isolated from different organs of identical patients, in the two cases examined, have significantly different karyotypes. Thus, while clones and/or parental strain from spleens of each patient clearly grouped together, they clustered separately from clones originating from the skin of the same patient. While changes in specific chromosome ploidy associated with parasite tropism were not identified, these results suggest that the aneuploidy patterns observed are a result of parasite origin (spleen or skin), and the differing conditions, perhaps temperature or host immune responses, to which the parasite is exposed.

SNP analysis, similar to MLMT [15], clearly shows that the Ethiopian *L*. *donovani* strains group, in large part, into two main populations, NE and SE, delineated by geography rather than clinical history (VL or HIV-VL co-infection; spleen or skin). Interestingly, the NE population appears to be more polymorphic than the SE population (Figs 2 and 3), reflecting the finding from MLMT data that inbreeding is higher for SE strains than NE strains [15]. In most cases, clones generated from an individual strain (GR363sp/sk, GR364sp/sk, GR383, GR356, AM560, etc), show more limited genetic polymorphism than that observed between strains, generally clustering together regardless of the method used for analysis (chromosome aneuploidy, SNPs or gene CNV). This was the case even when the patient strain(s) were isolated from different organs, such as skin and spleen, of the same HIV-VL co-infected patient, showing that the genotypes present in visceral organs can spread systemically in immunosuppressed patients to the skin where they get transmitted to sand flies. Only in one case, GR364sk/cl.II, did a cloned line fail to cluster with other clones generated from the patient strain. Instead, this clone grouped with SE strains both by SNP and CNV analysis. While contamination during cloning can’t be ruled out, parasites isolated from HIV co-infected patients have been shown to be more polymorphic than those isolated from patients with VL, and differences following patient treatment have been noted [56–58]. The chromosome karyotype and SNPs for this clone are distinct from all the SE strains suggesting that contamination, if it occurred, did not take place during generation of the cloned line.

SNP analysis also identified three strains, AM422, AM553 and LDS373bm, that didn’t fall, as expected, together with their respective geographic genotypes, SE and NE. LDS373bm and LDS373sp were isolated in parallel from different organs, bone marrow and spleen respectively, of the same HIV co-infected patient. The latter parasite (sp) is genetically similar to other parasite strains belonging to the NE population, while the former (bm) belongs to another genotype. These parasites are also different by k26 PCR typing of the HASP B repeat region [7]. This patient was apparently infected by at least two genotypes, with the genotype present in the bone marrow perhaps less virulent and only surviving in immune suppressed hosts. While several reports using MLMT, multilocus sequence typing and kDNA RFLP show that HIV-VL patients can be sequentially infected with genetically different parasites [58–60], to our knowledge this is the first time that an HIV-VL patient was shown to be simultaneously infected with two genetically different parasites. The amplicon (290 bp) seen for LDS373sp was typical of most NE strains examined (37/41), while for LDS373bm it was larger (410 bp), and observed in 4/41 NE strains, all HIV-VL co-infected patients. WGS of other parasites exhibiting the 410 bp amplicon was not carried out. It would be interesting to analyze more of these parasites and see if they form a separate genetic group. AM422 and AM553, both isolated in SE, fell outside the main NE and SE populations when SNPs were analyzed by two methods (Figs 2 and 3). Neither of these isolates was from patients co-infected with HIV. AM422 showed a k26 PCR amplicon typical of NE strains (290 bp) and originated in the Omo valley near the border with South Sudan, while AM553 had a unique k26 amplicon (360 bp). The unique SNP profiles for these two isolates suggest that additional genotypes are circulating in SE, perhaps a result of the more varied ecology in this region.

Gene CNV analysis also identifies differences that are typical of each geographic parasite population, NE and SE. Candidate genes that can be attributed to essential biological processes like drug resistance, virulence and parasite viability demonstrate differential CN among SE and NE strains and clones. While it is not clear which environmental and host factors resulted in the selective amplification of different genes in the NE and SE populations, many of them are essential genes important for parasite survival. Amplification or deletion of specific genes may give the parasites a growth advantage in the sand fly vector or human host. In the NE parasites, there are three times more copies of folate/biopterin transporter (FBT) genes on chromosome 10. *Leishmania* are folic acid auxotrophs, and *LDBPK_100400* is a homologue to the *Leishmania infantum* FT1 transporter that was defined as the main folate transporter in this *Leishmania* species. It is also known that FT1 transporter expression is upregulated in log phase of promastigote stage [34, 44], the stage found in sand fly midgut. Therefore, increased folate concentration in sand fly midgut may result in better parasite growth in the vector and provide the parasite with a better chance for survival and infection.

## Materials and methods

### Ethics statement

This study was conducted according to the Helsinki declaration, and was reviewed and approved by the Institutional Review Board (IRB), Medical Faculty, Addis Ababa University. Written informed consent was obtained from each adult study participant.

### Parasites origin and patient pathology

For this work 18 *L*. *donovani* strains isolated from 15 patients with VL in Ethiopia during the years 2009–2010 were selected for WGS (S1 Table). The selection was based on three criteria: 1) geographical origin (northern or southern Ethiopia); 2) Patient's pathology such as HIV/VL co-infection versus VL; and 3) source of parasites, skin versus spleen. Parasites were cultivated in M199 medium with supplements and rapidly frozen [7]. Additional Ethiopian (GR373 [7]) and Sudanese (LEMS3570, kindly provided by Prof. Patrick Bastein, National Reference Center of *Leishmania*, University Hospital Centre of Montpellier, France) strains were included in analysis of FT1 copy number, and are also listed in S1 Table. All parasites used in this study were characterized by ITS1 -, cpb—and k26—PCR ([7] and S4 Fig).

### Cloning

Eight patient strains were cloned prior to DNA extraction for WGS. The cloning procedure was carried out essentially as described [61].

### Next generation sequencing and sequence analysis

DNA was purified from *Leishmania* promastigotes that were harvested in their stationary growth stage in ~20ml M199 medium. DNA extraction was carried out as described by [7]

Genomic DNA was sheared into 400–600-base pair fragments by focused ultrasonication (Covaris Adaptive Focused Acoustics technology (AFA Inc., Woburn, USA)) and standard Illumina libraries were prepared. 100 base pair paired end reads were generated on the HiSeq 2000 v3 according to the manufacturer’s standard sequencing protocol [62]. Raw sequence data was deposited in the European Nucleotide Archive with the accession number ERP016010.

Sequence reads were mapped against the reference genome *Leishmania donovani*_21Apr2011 [16] using SMALT (version 0.7.4 https://sourceforge.net/projects/smalt/) to produce bam files. SMALT was used to index the reference using a kmer size of 13 and a step size of 2 (-k 13 -s 2) and the reads aligned. Reads were mapped if they had an identity of at least 90% to the reference genome and mapped uniquely to the genome. Reads in pairs were mapped independently, and marked as properly paired if they mapped in the correct orientation no more than 1.5 kb apart. PCR duplicate reads were identified using Picard v1.92(1464) and flagged as duplicates in the bam files.

### Prediction of chromosome aneuploidy

Aneuploidy was predicted based on whole chromosome median read coverage. For the normalization of median read coverage over all 36 chromosomes for a given strain, the average median coverage of four stable diploid chromosomes (chromosome 30, 32, 34 and 36) was calculated and taken as the mean read coverage for a diploid chromosome (*DC*^*mean*^). These four chromosomes have been previously shown to be diploid in almost all *L*. *donovani* isolates examined, and have been used for prediction of chromosome copy number [18, 20]. The predicted chromosome copy number is calculated as the fold change compared to *DC*^*mean*^. This prediction was applied to all 41 *L*. *donovani* sequences over 36 chromosomes, and saved in a 41*36 chromosome copy number matrix. We used R (*version 3*.*1*.*3*) [63] to evaluate of similarities and differences between strains and clones, and their chromosome aneuploidy patterns and computed a heat map over the chromosome copy number matrix with R *heatmap*.*2()* function from the *gplots* (version 2.17.0) R package. The numerical data for the creation of the heat map (Fig 1) is given in the supporting information (S2 Table). Median chromosome ploidy was used to compare average ploidy between NE and SE strains in Table 1. For isolates with multiple clones, average chromosome ploidy was calculated by determining the median somy of all the clones from an individual strain separately for each of the 36 chromosomes (S2 Table).

### SNP analysis

For the identification of population typical SNPs a procedure was implemented based on SNP and indel calling with the Genome Analysis Toolkit (GATK version 3.1) [64], VCFtools (version 0.1.12b) [65] and a custom R script based on Bioconductor packages (www.bioconductor.org) (Release (3.2)). The parameters for the first filtering procedure with GATK were set as follows: Emission confidence threshold > 10, Calling confidence threshold >50, read-depth > 500. A calculation of level of similarity between all 41 samples based on SNP profiles was computed with VCFtools, and processed with R based procedures into a similarity matrix. Finally, this matrix was visualized with *heatmap*.*2****()*** function from the *gplots* R package. Principal component analysis (PCA) following pruning of SNPs with high linkage disequilibrium was run using the Bioconductor package SNPRelate [26]. For the creation of unique SE or NE SNP profiles, only SNPs that were identified in >4 or >7 of the SE or NE clones and strains, respectively, were taken for final analysis. This protocol created a consensus SNP profile for each parasite population, as repetition of SNPs in more than 1/3 of the strains and clones for each population supported the accuracy of SNP calling and served as an additional quality control step. As a final step, unique SNP profiles were detected with VCFtool for each population. Unique SNPs present in coding regions that affect protein translation, namely non-synonymous or nonsense mutation(s) that change the amino acid or cause a stop codon were identified by a self-implemented R procedure based on Bioconductor packages. The mean absolute number of SNPs was compared between the SE and NE parasite populations, as well as to the Indian reference strain. Mean absolute SNPs for an individual patient isolate was calculated by averaging the SNPs from all clones of the respective strain. Variation in the number of SNPs between clones of an individual isolate was small, and % standard error varied from = 0.34–6.0 x 10^−3^, except for GR364sk (% s.e. = 0.064) when the atypical clone GR364sk/cl.II was included. In the absence of the atypical clone the % s.e. = 0.001.

### Prediction of gene copy number variation

The detection of copy number variations (CNV) and aberrations was done using the R- bioconductor [66] (www.bioconductor.org) package cn.mops (version 1.16.1) (Copy Number estimation by a Mixture Of PoissonS). cn.mops detected copy number variation as the normalized read depth variation at a certain genomic position over all 41 strains and clones. For that cn.mops calculated the read count matrices across all BAM files. For this analysis, the genomic window length was set to 5 Kbp and used as a sliding window for the prediction of genomic CNV. Therefore, a genomic position of certain strain or clone is considered as one with "CNV" if it shows a significant change in normalized read depth (> or < two i.e., diploidy) in a window length of 5 Kbp compared to other strains or clones at the same given genomic position. Further analysis genomic CNV that is localized in coding regions was carried out.

### Drug sensitivity

Parasites 2 x 10^6^ / ml were added in triplicate to 96-well plates and incubated with 0.5 mg/ml MTX (Sigma catalog number M8407) and in non-drug treated medium in final volume of 200 μl for 66 hours at 26°C. AlamarBlue (25 μl/well; AbD Serotec) was added and the viability was measured after four hours (λ_ex_ = 544; λ_em_ = 590; Fluroscan Ascent FL, Thermo) [67]. The percentage of killing was calculated as the fraction of fluorescence level of MTX treated wells compared to the non-drug treated wells.

### Quantitation of folate transporter 1 (FT1) gene copies by qPCR

The polycistronic folate/biopterin transporters (FT) on chromosome 10 have high DNA sequence similarity [29]. FT1 (*LDBPK_100400*) dual priming oligonucleotides (FT1-DPO) were used to specifically evaluate genomic CN for this gene by qPCR. FT1-DPO primers, forward FT1-DPO 5'-CGCCAGAACCCGAAGCCTGIIIIIGCACTGG-3' and reverse FT1-DPO 5'-GTTCATCACAGTCGCGATGAGTIIIIIAATCATTATG-3', were designed to include a polydeoxyinosine linker (IIIII) [30] that allows the specific primer annealing to the *LDBPK_100400* (FT1 ortholog) gene, and not to the other LD FT homologues. Specificity of the FT1-DPO PCR product was confirmed by cloning and sequencing of the amplicon. The *L*. *donovani* housekeeping gene alpha-tubulin was used for normalization. The qPCR conditions were the same for both FT1-DPO and housekeeping genes, and was carried out as follows: DNA (~10–20 ng) or no DNA control was added to HRM PCR Kit reaction mix (10 μl, QIAGEN GmbH, Germany) containing the FT1—DPO primers (1 μM each final concentration), and ultra-pure PCR-grade water (final volume 25 μl/PCR). Amplification conditions were as follows: 3 min denaturation at 95°C, followed by 40 cycles of denaturation 1 s at 95°C; annealing 20 s at 55°C; and extension 1 s at 65°C. HRM Ramping was carried out at 0.2°C/s from 65 to 95°C. HRM PCR and analysis were performed using a Rotor-Gene 6000 real-time PCR analyzer (Corbett Life Science, Australia). All reactions were carried out in duplicate and a negative-control reaction without parasite DNA was included in each experiment. For the calculation of the FT1 relative copy number (CN^rel^) the threshold (Ct) for all FT1 amplified samples were compared with their corresponding alpha-tubulin amplified samples as followed: CN^rel^ = 2^Ct(alphatubulin)-Ct(FT1)^. The CN^rel^ was further normalized based on the mean of the six lowest predicted relative CN. The mean value was considered as GCN = 1.

## Supporting information

S1 Fig Coding regions on chromosomes showing copy number variation for individual northern and southern Ethiopian *Leishmania donovani* strains. Genes that are not diploid are indicated by symbols.
(TIF)

S2 Fig Folate/biopterin transporter genes on chromosomes 10 and 35 showing regional differences in Copy Number Variation (CNV) associated with southern and northern Ethiopian *L*. *donovani* parasites. (TIF)

S3 Fig Growth inhibition of *Leishmania donovani* promastigotes from northern and southern Ethiopia by methotrexate as a function of folate concentration in the culture medium. A crosshatch titration on two parasite clones (SE—AM560/cl.IV and NE–GR383/cl.XIII) is shown below. Parasites were cultured in medium containing increasing concentrations of folic acid (FA) from 0 to 28.5 μg/ml (x-axis). To each of the culture conditions, increasing concentrations of methotrexate (MTX) from 0 to 1800 μg/ml (y-axis) was added, and parasite growth measured after 70 hours. The measurement of live promastigotes was carried out with alamarBlue^®^ assay as described by Shimony and Jaffe [67].
(TIF)

S4 Fig Characterization of *Leishmania donovani* strain LEMS3570 from Sudan by ITS1 -, cpb—and k26 –PCR. Don–*L*. *donovani* reference strain MHOM/SD/1962/1S Sudan cl2; Inf–*L*. *infantum* reference strain MHOM/TN/1980/IPT1; 3570 –LEMS3570. (TIF)

S1 Table Strains used in this study. (DOCX)

S2 Table A. Chromosome ploidy of the *Leishmania donovani* parasites analyzed by whole genome sequencing and used to compare strains from northern (NE) and southern (SE) Ethiopia. B. Chromosome ploidy of each strain and clone analyzed by whole genome sequencing.
(XLSX)

S3 Table A. Absolute number of SNPs in Ethiopian *Leishmania donovani* strains analyzed by whole genome sequencing. B. Absolute number of SNPs in each Ethiopian *Leishmania donovani* strain and clone analyzed by whole genome sequencing.
(XLSX)

S4 Table Data from PCA analysis of SNPs in 41 Ethiopian *Leishmania donovani* isolates. (DOCX)

S5 Table List of SNPs causing nonsynonymous and nonsense mutations that are unique for northern or southern Ethiopian *Leishmania donovani* populations. (XLS)

S6 Table Gene ontology enrichment in *Leishmania donovani* from Ethiopia based on SNPs unique to northern and southern parasite populations. (XLSX)

S7 Table REVIGO analysis of GO ontology terms for unique SNPs in northern and southern Ethiopian *Leishmania donovani* populations. (XLSX)

S8 Table Copy number variation of genes in northern and southern Ethiopian *Leishmania donovani*. (XLSX)

S9 Table Summary of genes showing differences in copy number variation between northern or southern Ethiopian *Leishmania donovani* populations. (XLSX)

S10 Table Predicted copy number variation of genes belonging to the folate/biopterin transporter family in Ethiopian *Leishmania donovani* strains. (DOCX)

S11 Table Folate transporter gene copy number is correlated with greater sensitivity to Methotrexate. (DOCX)

S12 Table Copy number variation (CNV) in the MAPK locus among SE and NE strain and clones. (DOCX)

S13 Table Haploid gene copy number variation in southern and northern Ethiopian *Leishmania donovani* populations. (XLS)

## References

[1] Alvar J, Velez ID, Bern C, Herrero M, Desjeux P, Cano J, et al Leishmaniasis worldwide and global estimates of its incidence. PLoS One. 2012;7(5):e35671 doi: 10.1371/journal.pone.0035671 22693548 10.1371/journal.pone.0035671 PMC3365071

[2] Anema A, Ritmeijer K. Treating HIV/AIDS and leishmaniasis coinfection in Ethiopia. Can Med Ass J. 2005;172(11):1434–5. 15911851 10.1503/cmaj.050511 PMC557972

[3] Alvar J, Aparicio P, Aseffa A, Den Boer M, Canavate C, Dedet JP, et al The relationship between leishmaniasis and AIDS: the second 10 years. Clin Microbiol Rev. 2008;21(2):334–59. doi: 10.1128/CMR.00061-07 18400800 10.1128/CMR.00061-07 PMC2292576

[4] Hurissa Z, Gebre-Silassie S, Hailu W, Tefera T, Lalloo DG, Cuevas LE, et al Clinical characteristics and treatment outcome of patients with visceral leishmaniasis and HIV co-infection in northwest Ethiopia. Trop Med Int Health. 2010;15(7):848–55. doi: 10.1111/j.1365-3156.2010.02550.x 20487426 10.1111/j.1365-3156.2010.02550.x

[5] Sundar S, Chakravarty J. Leishmaniasis: an update of current pharmacotherapy. Exp Opin Pharm. 2013;14(1):53–63. doi: 10.1517/14656566.2013.755515 . 23256501 10.1517/14656566.2013.755515

[6] Hailu A, Musa A, Wasunna M, Balasegaram M, Yifru S, Mengistu G, et al Geographical variation in the response of visceral leishmaniasis to paromomycin in East Africa: a multicentre, open-label, randomized trial. PLoS Negl Trop Dis. 2010;4(10):e709 doi: 10.1371/journal.pntd.0000709 21049059 10.1371/journal.pntd.0000709 PMC2964287

[7] Zackay A, Nasereddin A, Takele Y, Tadesse D, Hailu W, Hurissa Z, et al Polymorphism in the HASPB repeat region of East African *Leishmania donovani* strains. PLoS Negl Trop Dis. 2013;7(1):e2031 doi: 10.1371/journal.pntd.0002031 23358849 10.1371/journal.pntd.0002031 PMC3554577

[8] Hailu A, Gebre-Michael T, Berhe N, Balkew M. Leishmaniasis In: Berhane Y, Hailemariam D, Kloos H, editors. Epidemiology and Ecology of Health and Disease in Ethiopia. Addis Ababa: Shama Books; 2006 p. 615–34.

[9] Gebre-Michael T, Balkew M, Berhe N, Hailu A, Mekonnen Y. Further studies on the phlebotomine sandflies of the kala-azar endemic lowlands of Humera-Metema (north-west Ethiopia) with observations on their natural blood meal sources. Parasit Vectors. 2010;3(1):6 doi: 10.1186/1756-3305-3-6 20181077 10.1186/1756-3305-3-6 PMC2829606

[10] Elnaiem DE. Ecology and control of the sand fly vectors of Leishmania donovani in East Africa, with special emphasis on *Phlebotomus orientalis*. J Vector Ecol. 2011;36 Suppl 1:S23–31. 21366778 10.1111/j.1948-7134.2011.00109.x

[11] Gebre-Michael T, Lane RP. The roles of *Phlebotomus martini* and *P*.*celiae* (Diptera: Phlebotominae) as vectors of visceral leishmaniasis in the Aba Roba focus, southern Ethiopia. Med Vet Entomol. 1996;10(1):53–62. 8834743 10.1111/j.1365-2915.1996.tb00082.x

[12] Mauricio IL, Yeo M, Baghaei M, Doto D, Pratlong F, Zemanova E, et al Towards multilocus sequence typing of the *Leishmania donovani* complex: resolving genotypes and haplotypes for five polymorphic metabolic enzymes (ASAT, GPI, NH1, NH2, PGD). Int J Parasitol. 2006;36(7):757–69. doi: 10.1016/j.ijpara.2006.03.006 16725143 10.1016/j.ijpara.2006.03.006

[13] Kuhls K, Keilonat L, Ochsenreither S, Schaar M, Schweynoch C, Presber W, et al Multilocus microsatellite typing (MLMT) reveals genetically isolated populations between and within the main endemic regions of visceral leishmaniasis. Microbes Infect. 2007;9(3):334–43. doi: 10.1016/j.micinf.2006.12.009 17307010 10.1016/j.micinf.2006.12.009

[14] Jamjoom MB, Ashford RW, Bates PA, Chance ML, Kemp SJ, Watts PC, et al *Leishmania donovani* is the only cause of visceral leishmaniasis in East Africa; previous descriptions of *L*. *infantum* and "*L*. *archibaldi*" from this region are a consequence of convergent evolution in the isoenzyme data. Parasitol. 2004;129(Pt 4):399–409. 10.1017/s0031182004005955 15521628

[15] Gelanew T, Kuhls K, Hurissa Z, Weldegebreal T, Hailu W, Kassahun A, et al Inference of population structure of *Leishmania donovani* strains isolated from different Ethiopian visceral leishmaniasis endemic areas. PLoS Negl Trop Dis. 2011;4(11):e889. 10.1371/journal.pntd.0000889 PMC2982834 21103373

[16] Downing T, Imamura H, Decuypere S, Clark TG, Coombs GH, Cotton JA, et al Whole genome sequencing of multiple *Leishmania donovani* clinical isolates provides insights into population structure and mechanisms of drug resistance. Genome Res. 2011;21(12):2143–56. doi: 10.1101/gr.123430.111 22038251 10.1101/gr.123430.111 PMC3227103

[17] Downing T, Stark O, Vanaerschot M, Imamura H, Sanders M, Decuypere S, et al Genome-wide SNP and microsatellite variation illuminate population-level epidemiology in the *Leishmania donovani* species complex. Infect, Genet Evol. 2012;12(1):149–59. 22119748 10.1016/j.meegid.2011.11.005 PMC3315668

[18] Rogers MB, Hilley JD, Dickens NJ, Wilkes J, Bates PA, Depledge DP, et al Chromosome and gene copy number variation allow major structural change between species and strains of *Leishmania*. Genome Res. 2011;21(12):2129–42. doi: 10.1101/gr.122945.111 22038252 10.1101/gr.122945.111 PMC3227102

[19] Rogers MB, Downing T, Smith BA, Imamura H, Sanders M, Svobodova M, et al Genomic confirmation of hybridisation and recent inbreeding in a vector-isolated *Leishmania* population. PLoS Genetics. 2014;10(1):e1004092 doi: 10.1371/journal.pgen.1004092 24453988 10.1371/journal.pgen.1004092 PMC3894156

[20] Imamura H, Downing T, Van den Broeck F, Sanders MJ, Rijal S, Sundar S, et al Evolutionary genomics of epidemic visceral leishmaniasis in the Indian subcontinent. eLife. 2016;5. 10.7554/eLife.12613 PMC4811772 27003289

[21] Leprohon P, Fernandez-Prada C, Gazanion É, Monte-Neto R, Ouellette M. Drug resistance analysis by next generation sequencing in *Leishmania*. Int J Parasitol: Drugs Drug Res. 2015;5(1):26–35. 10.1016/j.ijpddr.2014.09.005 PMC4412915 25941624

[22] Zhang W-W, Matlashewski G. Screening *Leishmania donovani* Complex-Specific Genes Required for Visceral Disease In: Peacock C, editor. Parasite Genomics Protocols. Methods in Molecular Biology. 1201: Springer New York; 2015 p. 339–61. 10.1007/978-1-4939-1438-8_20 25388124

[23] Mondelaers A, Sanchez-Canete MP, Hendrickx S, Eberhardt E, Garcia-Hernandez R, Lachaud L, et al Genomic and Molecular Characterization of Miltefosine Resistance in *Leishmania infantum* Strains with Either Natural or Acquired Resistance through Experimental Selection of Intracellular Amastigotes. PLoS One. 2016;11(4):e0154101 doi: 10.1371/journal.pone.0154101 27123924 10.1371/journal.pone.0154101 PMC4849676

[24] Shaw CD, Lonchamp J, Downing T, Imamura H, Freeman TM, Cotton JA, et al In vitro selection of miltefosine resistance in promastigotes of *Leishmania donovani* from Nepal: genomic and metabolomic characterization. Mol Microbiol. 2016;99(6):1134–48. doi: 10.1111/mmi.13291 26713880 10.1111/mmi.13291 PMC4832254

[25] Llanes A, Restrepo CM, Vecchio GD, Anguizola FJ, Lleonart R. The genome of *Leishmania panamensis*: insights into genomics of the *L*. (*Viannia*) subgenus. Sci Rep. 2015;5. 10.1038/srep08550 PMC4338418 25707621

[26] Zheng X, Levine D, Shen J, Gogarten SM, Laurie C, Weir BS. A high-performance computing toolset for relatedness and principal component analysis of SNP data. Bioinformatics. 2012;28(24):3326–8. doi: 10.1093/bioinformatics/bts606 23060615 10.1093/bioinformatics/bts606 PMC3519454

[27] Supek F, Bo?njak M, kunca N, muc T. REVIGO Summarizes and Visualizes Long Lists of Gene Ontology Terms. PLoS ONE. 2011;6(7):e21800 doi: 10.1371/journal.pone.0021800 21789182 10.1371/journal.pone.0021800 PMC3138752

[28] El Fadili K, Messier N, Leprohon P, Roy G, Guimond C, Trudel N, et al Role of the ABC transporter MRPA (PGPA) in antimony resistance in *Leishmania infantum* axenic and intracellular amastigotes. Antimicrob Agents Chemother. 2005;49(5):1988–93. doi: 10.1128/AAC.49.5.1988-1993.2005 15855523 10.1128/AAC.49.5.1988-1993.2005 PMC1087671

[29] Ouameur AA, Girard I, Legare D, Ouellette M. Functional analysis and complex gene rearrangements of the folate/biopterin transporter (FBT) gene family in the protozoan parasite *Leishmania*. Mol Biochem Parasitol. 2008;162(2):155–64. doi: 10.1016/j.molbiopara.2008.08.007 18796316 10.1016/j.molbiopara.2008.08.007

[30] Chun JY, Kim KJ, Hwang IT, Kim YJ, Lee DH, Lee IK, et al Dual priming oligonucleotide system for the multiplex detection of respiratory viruses and SNP genotyping of CYP2C19 gene. Nucleic Acids Res. 2007;35(6):e40 doi: 10.1093/nar/gkm051 17287288 10.1093/nar/gkm051 PMC1874606

[31] Klambauer G, Schwarzbauer K, Mayr A, Clevert DA, Mitterecker A, Bodenhofer U, et al cn.MOPS: mixture of Poissons for discovering copy number variations in next-generation sequencing data with a low false discovery rate. Nucleic Acids Res. 2012;40(9):e69 doi: 10.1093/nar/gks003 22302147 10.1093/nar/gks003 PMC3351174

[32] Kaur K, Coons T, Emmett K, Ullman B. Methotrexate-resistant *Leishmania donovani* genetically deficient in the folate-methotrexate transporter. J Biol Chem. 1988;263(15):7020–8. 3366764

[33] Ellenberger TE, Beverley SM. Reductions in methotrexate and folate influx in methotrexate-resistant lines of *Leishmania major* are independent of R or H region amplification. J Biol Chem. 1987;262(28):13501–6. 3654626

[34] Richard D, Leprohon P, Drummelsmith J, Ouellette M. Growth phase regulation of the main folate transporter of *Leishmania infantum* and its role in methotrexate resistance. J Biol Chem. 2004;279(52):54494–501. doi: 10.1074/jbc.M409264200 15466466 10.1074/jbc.M409264200

[35] Mary C, Faraut F, Deniau M, Dereure J, Aoun K, Ranque S, et al Frequency of Drug Resistance Gene Amplification in Clinical *Leishmania* Strains. Internat J Microbiol. 2010;2010. 10.1155/2010/819060 PMC2913627 20706666

[36] Drini S, Criscuolo A, Lechat P, Imamura H, Skalický T, Rachidi N, et al Species- and Strain-Specific Adaptation of the HSP70 Super Family in Pathogenic Trypanosomatids. Genome Biol Evol. 2016;8(6):1980–95. doi: 10.1093/gbe/evw140 27371955 10.1093/gbe/evw140 PMC4943205

[37] Victoir K, Dujardin JC, de Doncker S, Barker DC, Arevalo J, Hamers R, et al Plasticity of gp63 gene organization in Leishmania (Viannia) braziliensis and Leishmania (Viannia) peruviana. Parasitol. 1995;111 (Pt 3):265–73. 10.1017/s0031182000081828 7567095

[38] Isnard A, Shio MT, Olivier M. Impact of *Leishmania* metalloprotease GP63 on macrophage signaling. Front Cell Infect Microbiol. 2012;2:72 doi: 10.3389/fcimb.2012.00072 22919663 10.3389/fcimb.2012.00072 PMC3417651

[39] Olivier M, Atayde VD, Isnard A, Hassani K, Shio MT. *Leishmania* virulence factors: focus on the metalloprotease GP63. Microbes Infect. 2012;14(15):1377–89. doi: 10.1016/j.micinf.2012.05.014 22683718 10.1016/j.micinf.2012.05.014

[40] Ellenberger TE, Beverley SM. Multiple drug resistance and conservative amplification of the H region in *Leishmania major*. J Biol Chem. 1989;264(25):15094–103. 2768255

[41] Marchini JF, Cruz AK, Beverley SM, Tosi LR. The H region HTBF gene mediates terbinafine resistance in *Leishmania major*. Mol Biochem Parasitol. 2003;131(1):77–81. 12967714 10.1016/s0166-6851(03)00174-9

[42] Ashutosh, Sundar S, Goyal N. Molecular mechanisms of antimony resistance in *Leishmania*. J Med Microbiol. 2007;56(2):143–53. 17244793 10.1099/jmm.0.46841-0

[43] Lakhal-Naouar I, Jardim A, Strasser R, Luo S, Kozakai Y, Nakhasi HL, et al *Leishmania donovani* Argininosuccinate Synthase Is an Active Enzyme Associated with Parasite Pathogenesis. PLoS Negl Trop Dis. 2012;6(10):e1849 doi: 10.1371/journal.pntd.0001849 23094117 10.1371/journal.pntd.0001849 PMC3475689

[44] Ouellette M, Drummelsmith J, El-Fadili A, Kundig C, Richard D, Roy G. Pterin transport and metabolism in *Leishmania* and related trypanosomatid parasites. Int J Parasitol. 2002;32(4):385–98. 11849635 10.1016/s0020-7519(01)00346-0

[45] Mittal MK, Rai S, Ashutosh, Ravinder, Guta S et al (2007) Characterization of natural antimony resistance in *Leishmania donovani* isolates. Amer J Trop Med Hyg 76: 681–688. 17426170

[46] Mukherjee A, Padmanabhan PK, Singh S, Roy G, Girard I, Chatterjee M, et al Role of ABC transporter MRPA, gamma-glutamylcysteine synthetase and ornithine decarboxylase in natural antimony-resistant isolates of *Leishmania donovani*. J Antimicrob Chemother. 2007;59(2):204–11. doi: 10.1093/jac/dkl494 17213267 10.1093/jac/dkl494

[47] Kazemi-Rad E, Mohebali M, Khadem-Erfan MB, Saffari M, Raoofian R, Hajjaran H, et al Identification of antimony resistance markers in *Leishmania tropica* field isolates through a cDNA-AFLP approach. Exp Parasitol. 2013;135(2):344–9. doi: 10.1016/j.exppara.2013.07.018 23928349 10.1016/j.exppara.2013.07.018

[48] Raymond F, Boisvert S, Roy G, Ritt JF, Legare D, Isnard A, et al Genome sequencing of the lizard parasite *Leishmania tarentolae* reveals loss of genes associated to the intracellular stage of human pathogenic species. Nucleic Acids Res. 2012;40(3):1131–47. doi: 10.1093/nar/gkr834 21998295 10.1093/nar/gkr834 PMC3273817

[49] Quilez J, Martinez V, Woolliams JA, Sanchez A, Pong-Wong R, Kennedy LJ, et al Genetic control of canine leishmaniasis: genome-wide association study and genomic selection analysis. PLoS One. 2012;7(4):e35349 doi: 10.1371/journal.pone.0035349 22558142 10.1371/journal.pone.0035349 PMC3338836

[50] Gelanew T, Cruz I, Kuhls K, Alvar J, Canavate C, Hailu A, et al Multilocus microsatellite typing revealed high genetic variability of *Leishmania donovani* strains isolated during and after a Kala-azar epidemic in Libo Kemkem district, northwest Ethiopia. Microbes Infect. 2011;13(6):595–601. doi: 10.1016/j.micinf.2011.02.003 21382503 10.1016/j.micinf.2011.02.003

[51] Boelaert M, El-Safi S, Hailu A, Mukhtar M, Rijal S, Sundar S, et al Diagnostic tests for kala-azar: a multi-centre study of the freeze-dried DAT, rK39 strip test and KAtex in East Africa and the Indian subcontinent. Trans R Soc Trop Med Hyg. 2008;102(1):32–40. doi: 10.1016/j.trstmh.2007.09.003 17942129 10.1016/j.trstmh.2007.09.003

[52] Ritmeijer K, Veeken H, Melaku Y, Leal G, Amsalu R, Seaman J, et al Ethiopian visceral leishmaniasis: generic and proprietary sodium stibogluconate are equivalent; HIV co-infected patients have a poor outcome. Trans R Soc Trop Med Hyg. 2001;95(6):668–72. 11816442 10.1016/s0035-9203(01)90110-5

[53] Zijlstra EE, el-Hassan AM. Leishmaniasis in Sudan. Post kala-azar dermal leishmaniasis. Trans R Soc Trop Med Hyg. 2001;95 Suppl 1:S59–76. 11370251 10.1016/s0035-9203(01)90219-6

[54] Sterkers Y, Lachaud L, Crobu L, Bastien P, Pages M. FISH analysis reveals aneuploidy and continual generation of chromosomal mosaicism in *Leishmania major*. Cell Microbiol. 2011;13(2):274–83. doi: 10.1111/j.1462-5822.2010.01534.x 20964798 10.1111/j.1462-5822.2010.01534.x

[55] Valdivia HO, Reis-Cunha JL, Rodrigues-Luiz GF, Baptista RP, Baldeviano GC, Gerbasi RV, et al Comparative genomic analysis of *Leishmania* (*Viannia*) *peruviana* and *Leishmania* (*Viannia*) *braziliensis*. BMC Genomics. 2015;16:715 doi: 10.1186/s12864-015-1928-z 26384787 10.1186/s12864-015-1928-z PMC4575464

[56] Chicharro C, Jimenez MI, Alvar J. Iso-enzymatic variability of *Leishmania infantum* in Spain. Ann Trop Med Parasitol. 2003;97 Suppl 1:57–64. 14678633 10.1179/000349803225002534

[57] Gramiccia M, Gradoni L, Troiani M. Heterogeneity among zymodemes of *Leishmania infantum* from HIV-positive patients with visceral leishmaniasis in south Italy. FEMS microbiology letters. 1995;128(1):33–8. 7744236 10.1111/j.1574-6968.1995.tb07496.x

[58] Cortes S, Mauricio IL, Kuhls K, Nunes M, Lopes C, Marcos M, et al Genetic diversity evaluation on Portuguese *Leishmania infantum* strains by multilocus microsatellite typing. Infect Genet Evol. 2014;26:20–31. doi: 10.1016/j.meegid.2014.04.023 24815728 10.1016/j.meegid.2014.04.023

[59] Gelanew T, Hailu A, Schonian G, Lewis MD, Miles MA, Yeo M. Multilocus sequence and microsatellite identification of intra-specific hybrids and ancestor-like donors among natural Ethiopian isolates of *Leishmania donovani*. Int J Parasitol. 2014;44(10):751–7. doi: 10.1016/j.ijpara.2014.05.008 24995620 10.1016/j.ijpara.2014.05.008 PMC4147965

[60] Morales MA, Cruz I, Rubio JM, Chicharro C, Canavate C, Laguna F, et al Relapses versus reinfections in patients coinfected with *Leishmania infantum* and human immunodeficiency virus type 1. J Infect Dis. 2002;185(10):1533–7. doi: 10.1086/340219 11992294 10.1086/340219

[61] Garin YJ, Meneceur P, Sulahian A, Derouin F. Microplate method for obtaining *Leishmania* clonal populations. J Parasitol. 2002;88(4):803–4. doi: 10.1645/0022-3395(2002)088[0803:MMFOLC]2.0.CO;2 12197138 10.1645/0022-3395(2002)088[0803:MMFOLC]2.0.CO;2

[62] Bronner IF, Quail MA, Turner DJ, Swerdlow H. Improved Protocols for Illumina Sequencing. Current protocols in human genetics / editorial board, Jonathan L Haines [et al]. 2014;80:18 2 1–42. 10.1002/0471142905.hg1802s80 26270174

[63] Team RC. R: A language and environment for statistical computing. Vienna, Austria: R Foundation for Statistical Computing; 2013 Available from: http://www.r-project.org/.

[64] McKenna A, Hanna M, Banks E, Sivachenko A, Cibulskis K, Kernytsky A, et al The Genome Analysis Toolkit: a MapReduce framework for analyzing next-generation DNA sequencing data. Genome Res. 2010;20(9):1297–303. doi: 10.1101/gr.107524.110 20644199 10.1101/gr.107524.110 PMC2928508

[65] Danecek P, Auton A, Abecasis G, Albers CA, Banks E, DePristo MA, et al The variant call format and VCFtools. Bioinformatics. 2011;27(15):2156–8. doi: 10.1093/bioinformatics/btr330 21653522 10.1093/bioinformatics/btr330 PMC3137218

[66] Gentleman RC, Carey VJ, Bates DM, Bolstad B, Dettling M, Dudoit S, et al Bioconductor: open software development for computational biology and bioinformatics. Genome Biol. 2004;5(10):R80 doi: 10.1186/gb-2004-5-10-r80 15461798 10.1186/gb-2004-5-10-r80 PMC545600

[67] Shimony O, Jaffe CL. Rapid fluorescent assay for screening drugs on *Leishmania* amastigotes. J Microbiol Methods. 2008;75(2):196–200 doi: 10.1016/j.mimet.2008.05.026 18573286 10.1016/j.mimet.2008.05.026

