# Supplementary figures and images for: Genome wide comparison of Ethiopian Leishmania donovani strains reveals differences potentially related to parasite survival
Source: PLoS Genet. 2018 Jan 9;14(1):e1007133. doi: 10.1371/journal.pgen.1007133 (PMC5777657; doi:10.1371/journal.pgen.1007133)

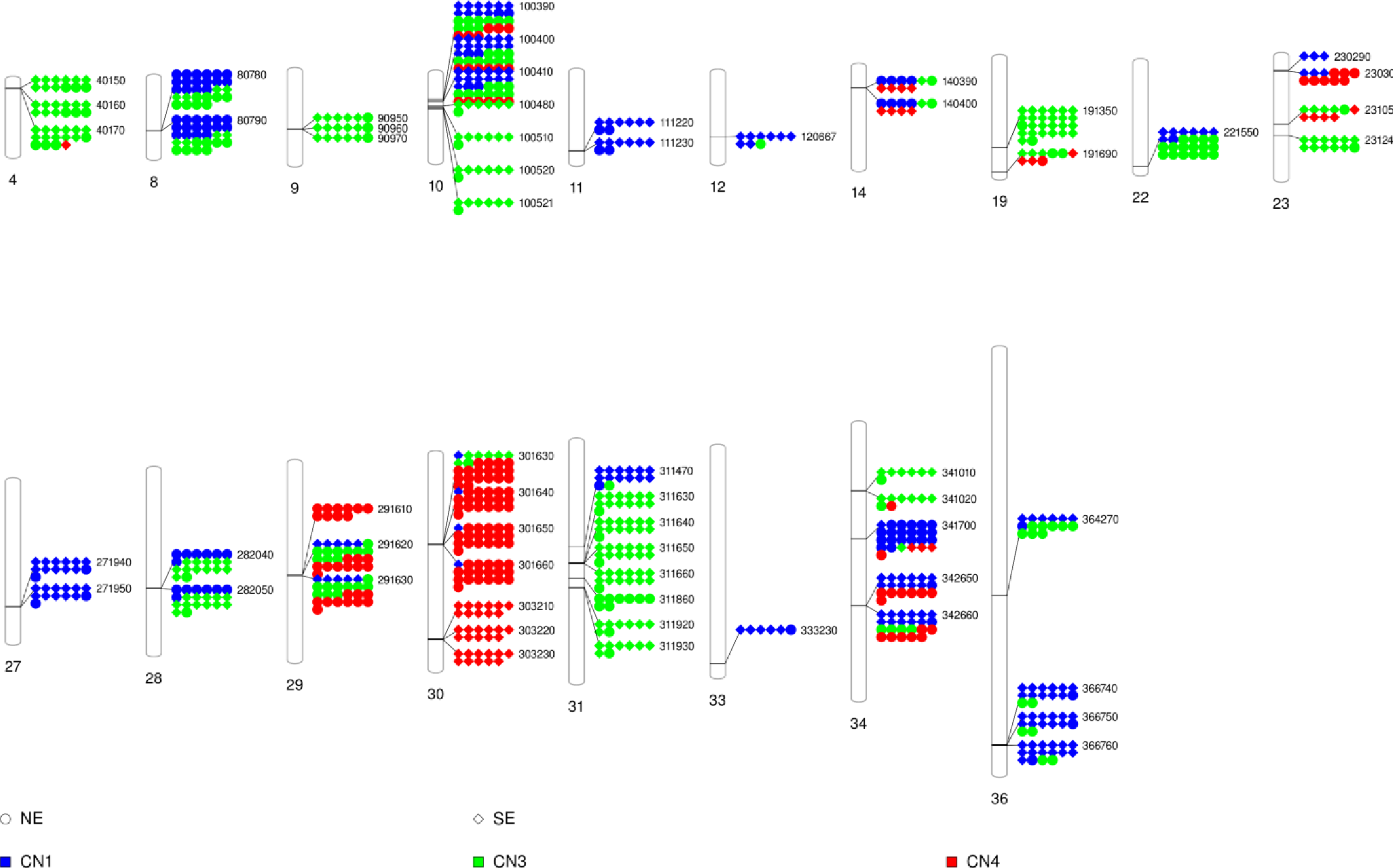

Supplement: S1 Fig — Genes that are not diploid are indicated by symbols. (TIF) [file pgen.1007133.s001.tif]

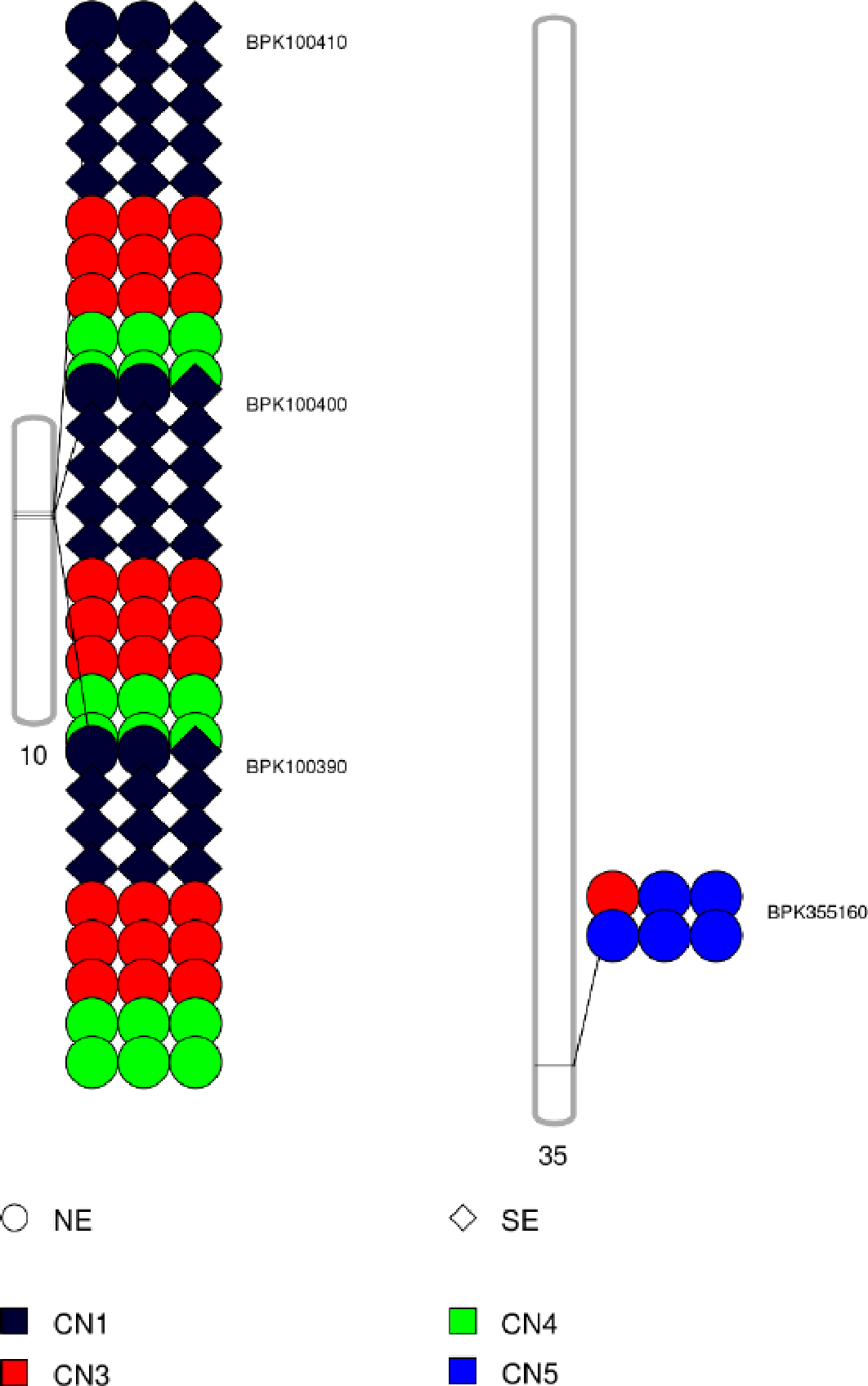

Supplement: S2 Fig — (TIF) [file pgen.1007133.s002.tif]

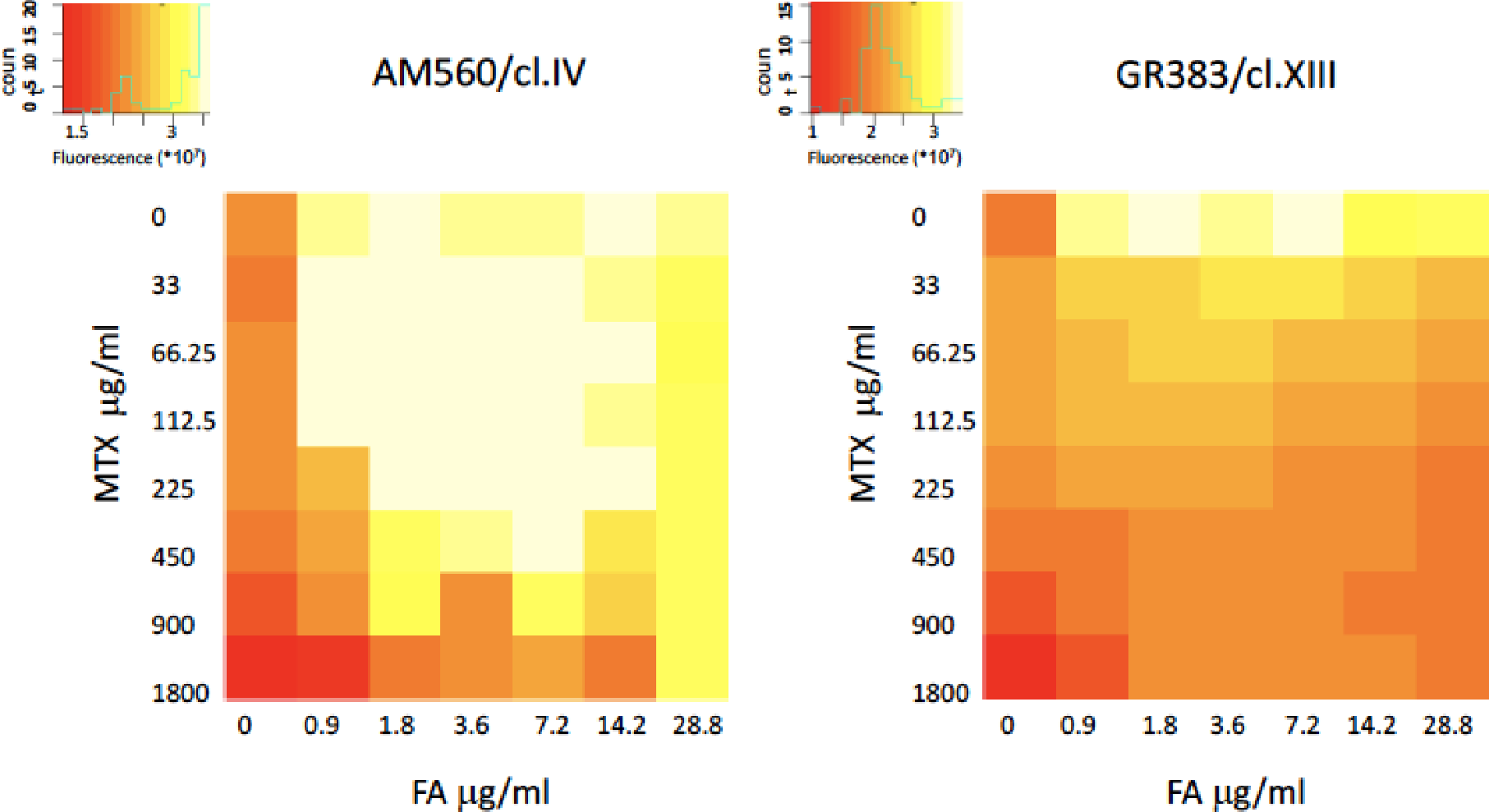

Supplement: S3 Fig — A crosshatch titration on two parasite clones (SE—AM560/cl.IV and NE–GR383/cl.XIII) is shown below. Parasites were cultured in medium containing increasing concentrations of folic acid (FA) from 0 to 28.5 μg/ml (x-axis). To each of the culture conditions, increasing concentrations of methotrexate (MTX) from 0 to 1800 μg/ml (y-axis) was added, and parasite growth measured after 70 hours. The measurement of live promastigotes was carried out with alamarBlue® assay as described by Shimony and Jaffe [67]. (TIF) [file pgen.1007133.s003.tif]

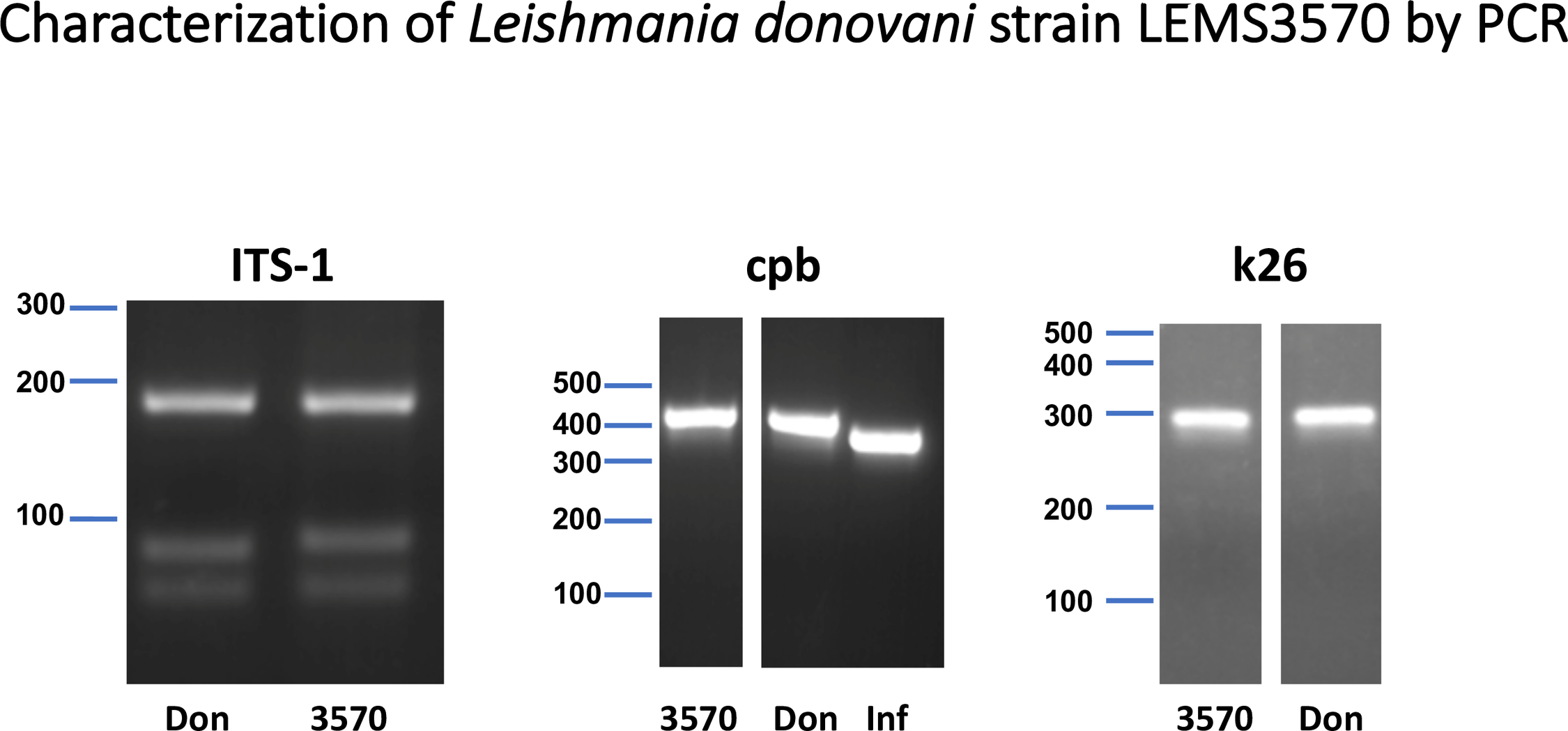

Supplement: S4 Fig — (TIF) [file pgen.1007133.s004.tif]
