# Supplementary material for: Genome wide comparison of Ethiopian Leishmania donovani strains reveals differences potentially related to parasite survival
Source: PLoS Genet. 2018 Jan 9;14(1):e1007133. doi: 10.1371/journal.pgen.1007133 (PMC5777657; doi:10.1371/journal.pgen.1007133)
Supplement: S1 Table — (DOCX) [file pgen.1007133.s005.docx]

Table S1. Strains used in this study.

| **WHO Code** | **Analyzed by WGS^a^** | **Pathology** | **k26 – PCR (bp)^c^** | **Likely infection location^d^** |
| --- | --- | --- | --- | --- |
| MHOM/ET/2009/AM421 | P | HIV-VL/ sp | 450 | Konso, SE |
| MHOM/ET/2009/AM422 | P | HIV-VL/ sp | 290 | Omo Valley, SE |
| MHOM/ET/2010/AM546 | P | VL/ sp | 450 | Konso, SE |
| MHOM/ET/2010/AM548 | P | VL/ sp | 450 | Konso, SE |
| MHOM/ET/2010/AM551 | P | VL/ sp | 450 | Konso, SE |
| MHOM/ET/2010/AM552 | P | VL/ sp | 450 | Negele-Borena, SE |
| MHOM/ET/2010/AM553 | P | VL/ sp | 360 | Negele-Borena, SE |
| MHOM/ET/2010/AM554 | P | VL/ sp | 450 | Negele-Borena, SE |
| MHOM/ET/2010/AM560 | cl.I | VL/ sp | 450 | Konso, SE |
| MHOM/ET/2010/AM560 | cl.II | VL/ sp | 450 | Konso, SE |
| MHOM/ET/2010/AM560 | cl.III | VL/ sp | 450 | Konso, SE |
| MHOM/ET/2010/AM560 | cl.IV | VL/ sp | 450 | Konso, SE |
| MHOM/ET/2010/AM563 | cl.I | VL/ sp | 450 | Negele-Borena, SE |
| MHOM/ET/2009/GR356 | cl.I | VL/ sp | 290 | N/Gondar - Metema, NE |
| MHOM/ET/2009/GR356 | cl.II | VL/ sp | 290 | N/Gondar - Metema, NE |
| MHOM/ET/2009/GR356 | cl.III | VL/ sp | 290 | N/Gondar - Metema, NE |
| MHOM/ET/2009/GR356 | cl.IV | VL/ sp | 290 | N/Gondar - Metema, NE |
| MHOM/ET/2009/GR356 | cl.V | VL/ sp | 290 | N/Gondar - Metema, NE |
| MHOM/ET/2009/GR356 | cl.VI | VL/ sp | 290 | N/Gondar - Metema, NE |
| MHOM/ET/2009/GR356 | cl.VII | VL/ sp | 290 | N/Gondar - Metema, NE |
| MHOM/ET/2009/GR356 | cl.VIII | VL/ sp | 290 | N/Gondar - Metema, NE |
| MHOM/ET/2009/GR356 | cl.XI | VL/ sp | 290 | N/Gondar - Metema, NE |
| MHOM/ET/2010/GR363sp | cl.I | HIV-VL/ sp | 290 | Humera, NE |
| MHOM/ET/2010/GR363sp | cl.II | HIV-VL/ sp | 290 | Humera, NE |
| MHOM/ET/2010/GR363sp | cl.X | HIV-VL/ sp | 290 | Humera, NE |
| MHOM/ET/2010/GR363sk | cl.I | HIV-VL/sk | 290 | Humera, NE |
| MHOM/ET/2010/GR363sk | cl.II | HIV-VL/sk | 290 | Humera, NE |
| MHOM/ET/2010/GR363sk | cl.X | HIV-VL/sk | 290 | Humera, NE |
| MHOM/ET/2010/GR364sp | cl.I | HIV-VL/ sp | 290 | Humera, NE |
| MHOM/ET/2010/GR364sp | cl.II | HIV-VL/ sp | 290 | Humera, NE |
| MHOM/ET/2010/GR364sp | cl.III | HIV-VL/ sp | 290 | Humera, NE |
| MHOM/ET/2010/GR364sk | P | HIV-VL/sk | 290 | Humera, NE |
| MHOM/ET/2010/GR364sk | cl.I | HIV-VL/sk | 290 | Humera, NE |
| MHOM/ET/2010/GR364sk | cl.II | HIV-VL/sk | 450 | Humera, NE |
| MHOM/ET/2010/GR383 | cl.I | VL/ sp | 290 | Humera, NE |
| MHOM/ET/2010/GR383 | cl.II | VL/ sp | 290 | Humera, NE |
| MHOM/ET/2010/GR383 | cl.X | VL/ sp | 290 | Humera, NE |
| MHOM/ET/2010/GR383 | cl.XII | VL/ sp | 290 | Humera, NE |
| MHOM/ET/2010/GR383 | cl.XIII | VL/ sp | 290 | Humera, NE |
| MHOM/ET/2009/LDS373bm | P | HIV-VL/ bm | 410 | N/Gondar/Humera, NE |
| MHOM/ET/2009/LDS373sp | P | HIV-VL/sp | 290 | N/Gondar/Humera, NE |
| MHOM/ET/2010/GR379 | P | VL/sp | 290 | Metema, NE |
| MHOM/SD/98/LEMS3570 | P | PKDL/sk | 290 | Barbar El Fugara, Sudan |

^a^P-parental strain, cl-clone; ^b^VL - visceral leishmaniasis, HIV/VL – HIV - VL co-infection, sp – spleen isolate, sk – skin isolate and bm – bone marrow; ^c^Zackay et al. (2013) PLoS NTD 7:e2031; ^d^NE-northern Ethiopia, SE-southern Ethiopia
