# Supplementary material for: Genome wide comparison of Ethiopian Leishmania donovani strains reveals differences potentially related to parasite survival
Source: PLoS Genet. 2018 Jan 9;14(1):e1007133. doi: 10.1371/journal.pgen.1007133 (PMC5777657; doi:10.1371/journal.pgen.1007133)
Supplement: S4 Table — (DOCX) [file pgen.1007133.s008.docx]

Table S4. Data from PCA analysis of SNPs in 41 Ethiopian Leishmania donovani isolates. Working space: 41 samples, 6282 SNPs with LD threshold= 0.9.

**sample.id pop EV1 EV2**

LDS373SP NE -0.034 0.020

LDS373SP NE -0.046 0.018

GR383/cl.XIII NE -0.039 0.036

GR383/cl.XII NE -0.047 0.032

GR383/cl.XII NE 0.002 -0.013

GR383/cl.X NE -0.047 0.033

GR383/cl.X NE -0.031 0.021

GR383/cl.II NE -0.048 0.033

GR383/cl.I NE -0.046 0.032

GR364SP/cl.III NE -0.035 0.021

GR364SP/cl.II NE -0.034 0.021

GR364SK/cl.II NE 0.002 0.003

GR364SK/cl.I NE -0.032 0.019

GR363SP/cl.X NE -0.045 0.038

GR363SP/cl.II NE -0.044 0.038

GR363SP/cl.I NE -0.045 0.035

GR363SK/cl.X NE -0.045 0.038

GR363SK/cl.II NE -0.045 0.037

GR363SK/cl.I NE -0.046 0.037

GR356/cl.XI NE -0.055 0.019

GR356/cl.VIII NE -0.054 0.023

GR356/cl.VII NE -0.050 0.018

GR356/cl.VI NE -0.048 0.024

GR356/cl.V NE -0.055 0.026

GR356/cl.IV NE -0.050 0.018

GR356/cl.III NE -0.053 0.024

GR356/cl.II NE -0.049 0.020

GR356/cl.I NE -0.052 0.024

AM563/cl.I SE -0.001 0.002

AM560/cl.IV SE -0.001 0.000

AM560/cl.III SE -0.001 0.000

AM560/cl.II SE 0.000 0.001

AM560/cl.I  SE 0.000 -0.001

AM554 SE 0.000 0.000

AM553 SE 0.945 0.265

AM552 SE 0.000 -0.002

AM551 SE 0.000 -0.004

AM548 SE 0.001 0.000

AM546 SE -0.001 -0.001

AM422 SE 0.229 -0.954

AM421 SE 0.000 -0.001
