# Supplementary material for: Genome wide comparison of Ethiopian Leishmania donovani strains reveals differences potentially related to parasite survival
Source: PLoS Genet. 2018 Jan 9;14(1):e1007133. doi: 10.1371/journal.pgen.1007133 (PMC5777657; doi:10.1371/journal.pgen.1007133)
Supplement: S10 Table — (DOCX) [file pgen.1007133.s014.docx]

Table S10. Predicted copy number variation of genes belonging to the folate/biopterin transporter family in Ethiopian *L. donovani* strains.

|  | NE | | SE | |
| --- | --- | --- | --- | --- |
| Gene | Strain | GCN^#^ | Strain | GCN |
| LdBPK_060310 | all | 2 | all | 2 |
| LdBPK_061320 | all | 2 | all | 2 |
| LdBPK_100360 | all | 2 | all | 2 |
| LdBPK_100370 | all | 2 | all | 2 |
| LdBPK_100380 | all | 2 | AM551Others | 12 |
| LdBPK_100390 | S3570GR364skGR364sk/cl.IGR364sp/cl.IGR364sp/cl.IIGR364sp/cl.IIIGR356/cl.IGR356/cl.IIGR356/cl.IIIGR356/cl.IVGR356/cl.VGR356/cl.VIGR356/cl.VIIGR356/cl.VIIIGR356/cl.XIGR364sk/cl.II*GR373bm*Others | 444444333333333112 | AM421AM422AM548AM551AM552AM553AM554AM560/cl.IAM560/cl.IIAM560/cl.IIIAM560/cl.IVOthers | 111111111112 |
| LdBPK_100400 (FT1) | S3570GR364skGR364sk/cl.IGR364sp/cl.IGR364sp/cl.IIGR364sp/cl.IIIGR356/cl.IGR356/cl.IIGR356/cl.IIIGR356/cl.IVGR356/cl.VGR356/cl.VIGR356/cl.VIIGR356/cl.VIIIGR356/cl.XIGR364sk/cl.IIGR373bmOthers | 444444333333333112 | AM421AM422AM546AM548AM551AM552AM553AM554AM560/cl.IAM560/cl.IIAM560/cl.IIIAM560/cl.IVAM563/cl.I | 1111111111111 |
| LdBPK_100410 | S3570GR364skGR364sk/cl.IGR364sp/cl.IGR364sp/cl.IIGR364sp/cl.IIIGR356/cl.IGR356/cl.IIGR356/cl.IIIGR356/cl.IVGR356/cl.VGR356/cl.VIGR356/cl.VIIGR356/cl.VIIIGR356/cl.XIGR364sk/cl.IIGR373bmOthers | 444444333333333112 | AM421AM422AM546AM548AM551AM552AM553AM554AM560/cl.IAM560/cl.IIAM560/cl.IIIAM560/cl.IVAM563/cl.I | 1111111111111 |
| LdBPK_100420 (FT5?) | all | 2 | all | 2 |
| LdBPK_100450 | all | 2 | all | 2 |
| LdBPK_141440 | all | 2 | all | 2 |
| LdBPK_190870 | all | 2 | all | 2 |
| LdBPK_355160(BT1) | GR373bmGR383/cl.IGR383/cl.IIGR383/cl.XGR383/cl.XIIGR383/cl.XIIIOthers | 3555552 | all | 2 |

### Footnotes: ^#^GCN - gene copy number for an individual strain or the average gene copy number for all clones derived from each strain.* GR364sk/cl.II and GR373bm* group with SE strains by SNP analysis.
