# Supplementary material for: Genome wide comparison of Ethiopian Leishmania donovani strains reveals differences potentially related to parasite survival
Source: PLoS Genet. 2018 Jan 9;14(1):e1007133. doi: 10.1371/journal.pgen.1007133 (PMC5777657; doi:10.1371/journal.pgen.1007133)
Supplement: S11 Table — (DOCX) [file pgen.1007133.s015.docx]

Table S11. Folate transporter gene copy number is correlated with greater sensitivity to Methotrexate.

| Strain/clone† | Origin | GeneID:CN* | % Dead ± s.e.** |
| --- | --- | --- | --- |
| AM563/cl.I | SE | LdBPK_100400:CN1 LdBPK_100410:CN1 | 30 ± 14 |
| AM560/cl.I | SE | LdBPK_100390:CN1LdBPK_100400:CN1LdBPK_100410:CN1 | 6 ± 5 |
| AM560/cl.IV | SE | LdBPK_100390:CN1LdBPK_100400:CN1LdBPK_100410:CN1 | 9 ± 2 |
| AM551 | SE | LdBPK_100380:CN1LdBPK_100390:CN1LdBPK_100400:CN1LdBPK_100410:CN1 | 13 ± 7 |
| GR356/cl.IV | NE | LDBPK_100390:CN3LDBPK_100400:CN3LDBPK_100410:CN3 | 50 ± 3 |
| GR363sp/cl.X | NE | Diploid for all FTs | 42 ± 15 |
| GR383/cl.XIII | NE | LDBPK_355160:CN5 | 60 ± 2 |
| GR364sp/cl.III | NE | LDBPK_100390:CN4LDBPK_100400:CN4LDBPK_100410:CN4 | 78 ± 4 |

†Strains from southern Ethiopia (SE) have codes beginning with AMxxx, where xxx represents the strain number. Strains from northern Ethiopia (NE) have codes beginning with either GRxxx, where xxx represents the strain number. *CN predicted by cn.mops. **%Dead - percentage parasites killed following incubation with 0.5mg/ml methotrexate as described in Material and Methods. LdBPK_100400 = FT1; LdBPK_355160 = BT1. Average of n=3 independent experiments ± s.e.

.
