# Supplementary material for: Genome wide comparison of Ethiopian Leishmania donovani strains reveals differences potentially related to parasite survival
Source: PLoS Genet. 2018 Jan 9;14(1):e1007133. doi: 10.1371/journal.pgen.1007133 (PMC5777657; doi:10.1371/journal.pgen.1007133)
Supplement: S12 Table — (DOCX) [file pgen.1007133.s016.docx]

Table S12. Copy number variation (CNV) in the MAPK locus among SE and NE strain and clones.

|  | Average CN (No. strains & clones) | |
| --- | --- | --- |
| Gene | SE* | NE |
| LDBPK_366740 | 1 (12/15) | 2 (25/27) |
| LDBPK_366750 | 1 (12/15) | 2 (25/27) |
| LDBPK_366760 | 1 (14/15) | 2 (22/27) |
| LDBPK_366770 | 2 (14/15) | 2 (26/27) |

*SE includes all SE and SE-like NE strains and clones.
